# Mass spectrometry dataset of conventional and organic tempe before and after *in vitro* digestion

**DOI:** 10.1016/j.dib.2025.111821

**Published:** 2025-06-23

**Authors:** Nurul Syahidah Mio Asni, Norazlan Mohmad Misnan, Ahmed Mediani, Ivana Nur Allisya Rozlan, Nurul Amalia Zahari, Syarul Nataqain Baharum, Nurkhalida Kamal

**Affiliations:** a Institute of Systems Biology (INBIOSIS), Universiti Kebangsaan Malaysia (UKM), Bangi 43600, Malaysia; b Herbal Medicine Research Centre, Institute for Medical Research, National Institutes of Health, Ministry of Health Malaysia, Shah Alam 40170, Selangor, Malaysia

**Keywords:** Metabolomics, Metabolites, Fermented food, Digestion

## Abstract

Tempe is a superior plant-based protein source that provides a diverse array of nutritional benefits as a result of the presence of bioactive metabolites. Nevertheless, there is a scarcity of information regarding the metabolomics profile between organic and conventional tempe and the fate of these metabolites after *in vitro* digestion. This report examines the metabolomic profile of soybean as raw material and tempe prior to and following the *in vitro* digestion process. We obtained a comprehensive set of metabolomic data using ultra-high-performance liquid chromatography coupled with high-resolution mass spectrometry (UHPLC-HRMS). The metabolomics dataset organized into Excel sheets and structured according to polarity, mass to charge ratio (m/z), retention time, feature name, biological replicates and controls. This data offers preliminary insights into the metabolite profile of tempe samples, encompassing source material soybean, tempe, and tempe digesta.

Specifications Table

**Table:** 

Subject	Biological Sciences
Specific subject area	Metabolomics
Type of data	Figure Raw data
Data collection	Ultra-high-performance liquid chromatography coupled with high-resolution mass spectrometry were acquired from Thermo Fisher Scientific UltiMate 3000 UHPLC system, equipped with the WPS-3000RS autosampler, TCC-3000RS column compartment, and RS pump coupled with a heat electrospray ionization (HESI). The separations were conducted using an ACQUITY UPLC® BEH C18 analytical column (2.1 mm × 100 mm; particle size, 1.7 μm). HRMS data were obtained using a Thermo Scientific Q-Exactive Orbitrap mass spectrometer, which was managed by the Xcalibur 3.0.63 software
Data source location	Institute of Systems Biology (INBIOSIS), Universiti Kebangsaan Malaysia, 43600 UKM Bangi, Selangor Malaysia.
Data accessibility	The complete dataset is accessible at Mendeley Data Repository, DOI: 10.17632/ty2v38d9p6.1 https://data.mendeley.com/datasets/ty2v38d9p6/1
Related research article	Mio Asni, N. S., Surya, R., Mohmad Misnan, N., Lim, S. J., Ismail, N., Sarbini, S. R., & Kamal, N. (2024). Metabolomics insights of conventional and organic tempe during *in vitro* digestion and their antioxidant properties and cytotoxicity in HCT-116 cells. *Food research international*, 195, 114951. [1] 10.1016/j.foodres.2024.114951

## 1 Value of the Data

• The comprehensive metabolomics data illuminates the nutritional qualities of both organic and conventional tempe, facilitating a better understanding of the health benefits associated with these protein sources and their bioactive metabolites.
• By evaluating the changes in the metabolite profile before and after *in vitro* digestion, this research provides crucial insights into how bioactive compounds are altered after digestion, which may affect their bioavailability and overall health impacts.
• The organization of metabolomics data allows for a detailed comparative analysis between the raw soybean and its tempe form, offering valuable information for further research on the effects of fermentation processes and agricultural practices on nutrient composition in plant-based foods.

## 2 Background

Our previous study addresses the growing interest in organic soybean products and the understanding of how agricultural practices impact the bioavailability of bioactive compounds in fermented foods, specifically tempe. As dietary choices increasingly focus on health benefits, it is also essential to investigate the metabolomic changes that occur after digestion and how these may influence potential health outcomes. By examining conventional and organic tempe through metabolomics analysis, this study provides valuable insights into the digestive bioavailability of bioactive compounds, supporting a better understanding of the relation between food processing, nutrient absorption, and health effects. This dataset is intended to provide a fundamental resource for comparative metabolomics in fermented foods, offering valuable support to the food metabolomics research.

## 3 Data Description

• **Neg Raw Data mzML:** A total of 24 raw file from negative ionisation in mzML format from 6 different sample groups (CS, OS, CT, OT, CTD, OTD) with 4 replicates each.
• **Pos Raw Data mzML:** A total of 24 raw file from positive ionisation in mzML format from 6 different sample groups (CS, OS, CT, OT, CTD, OTD) with 4 replicates each.
• **Combine Polarity Data_Macro DNP Prosessed.xlsx** A table containing pre-processed dataset with key information (m/z, retention time, feature names) from each feature from both positive and negative ionisation.
• **Neg Data_Tempe Study.xlsx** A table containing pre-processed dataset with key information (m/z, retention time, feature names) from each feature from negative ionisation.
• **Pos Data_Tempe Study.xlsx** A table containing pre-processed dataset with key information (m/z, retention time, feature names) from each feature from positive ionisation.

**Fig. 1:**
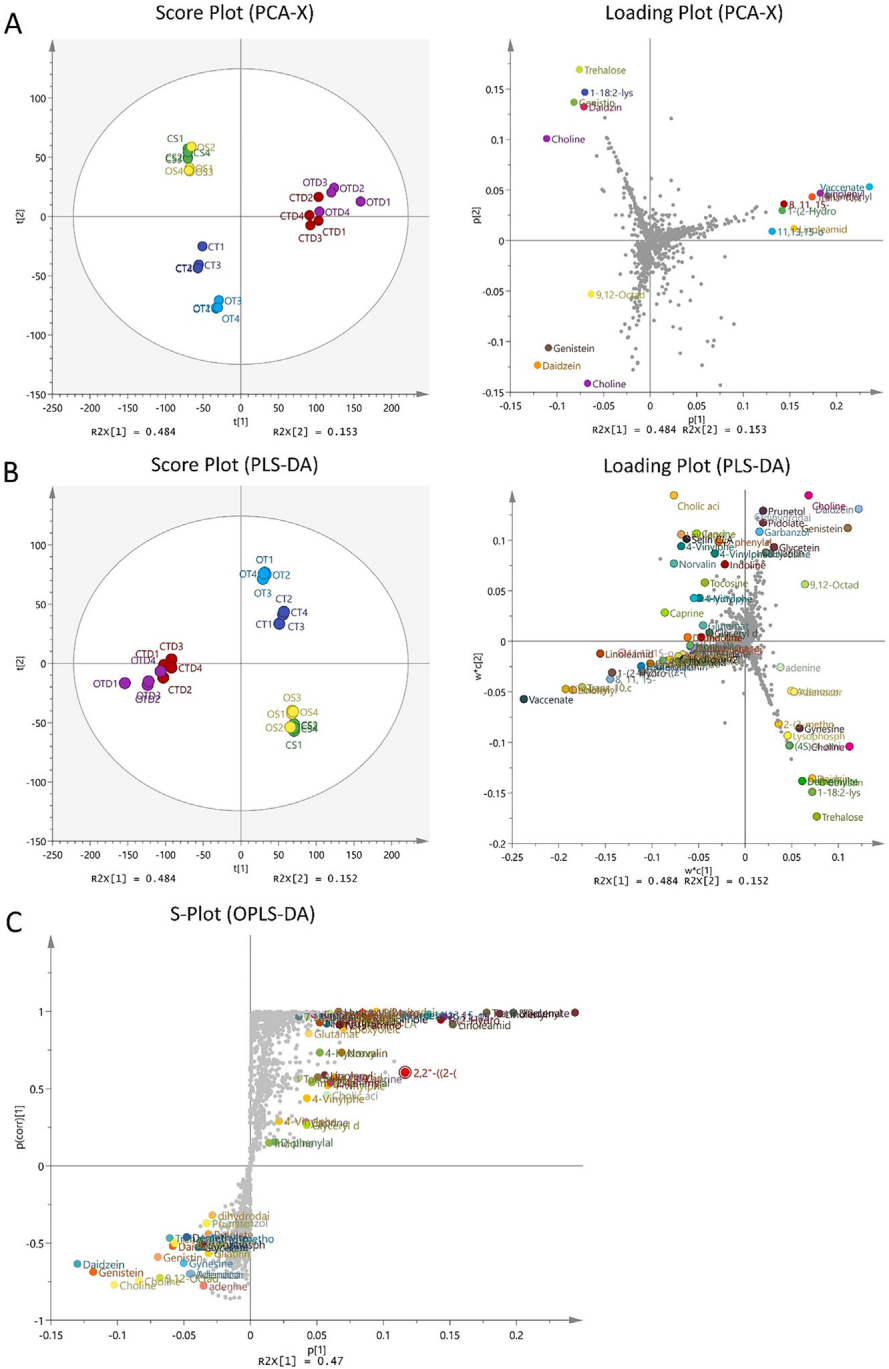
Multivariate analysis of metabolites and heatmap of isoflavones in all samples. **(A)** Principal component analysis (PCA) score plot derived from the LC–HR-MS profiles of different sample groups: Conventional Soybean (CS), Organic Soybean (OS), Conventional Tempe (CT), Organic Tempe (OT), Conventional Tempe Digesta (CTD) and Organic Tempe Digest (OTD). **(B)** Partial Least Squared Discriminant Analysis (PLS-DA) loading plot highlighting metabolites with Variable Importance in Projection (VIP) scores greater than 1 (VIP > 1). **(C)** An S-plot showing the metabolites according to the importance of variables which are responsible for driving the separation of samples. This figure was reproduced from Mio Asni et al. (2024), *Food Research International*, 195, 114951 [1]. DOI: 10.1016/j.foodres.2024.114951. Copyright (2024) by Elsevier.

**Fig. 2:**
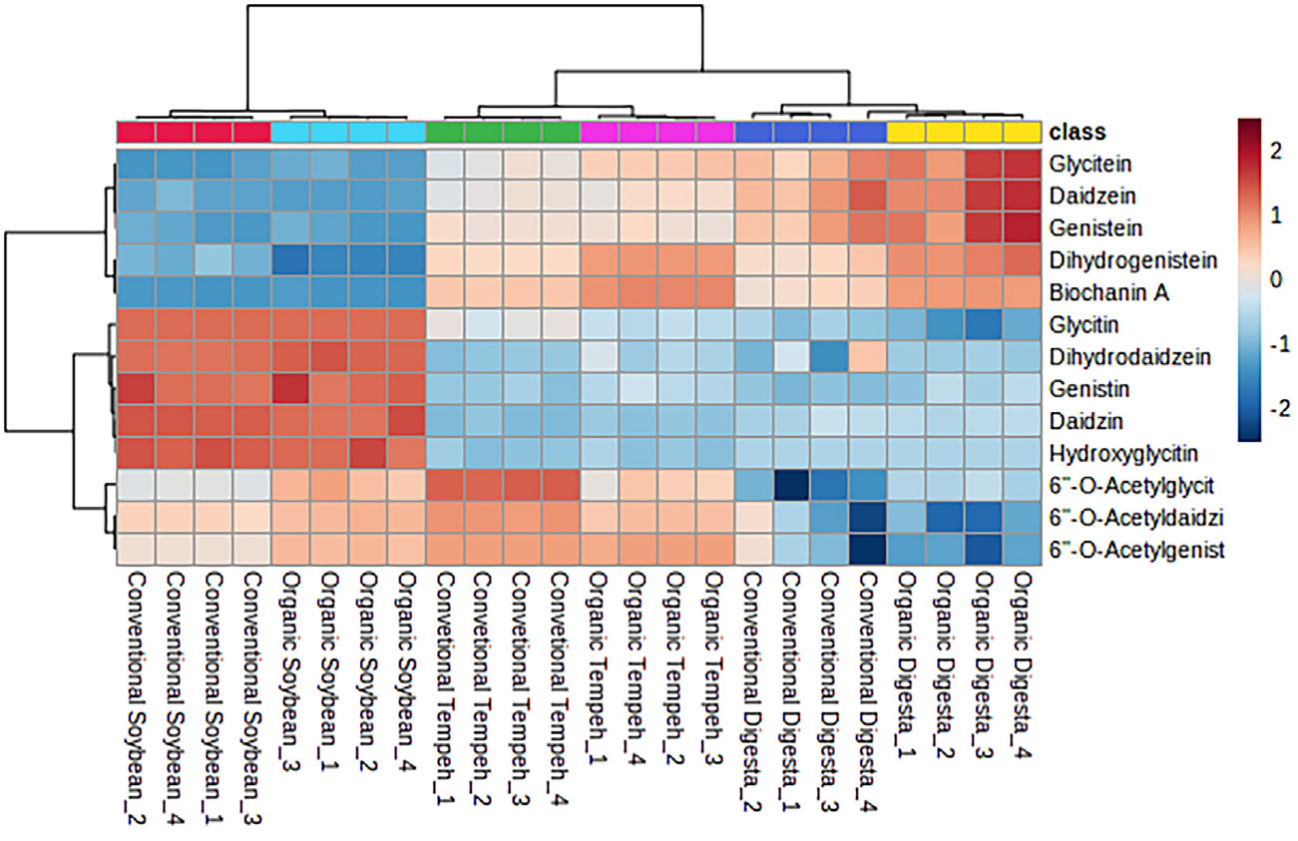
Heatmap of the different sample groups against isoflavones was visualised using MetaboAnalyst 5.0 software. This figure was reproduced from Mio Asni et al. (2024), *Food Research International*, 195, 114951 [1]. DOI: 10.1016/j.foodres.2024.114951. Copyright (2024) by Elsevier.

Thermo Scientific Q-Exactive Orbitrap mass spectrometer with positive and negative ion modes were used to generate mass spectra, and Ultra-High Performance Liquid Chromatography–Tandem Mass Spectrometry (UHPLC-MS/MS) was used to analyze metabolites. Score plots and loading plots from principal component analysis (PCA) and partial least squares discriminant analysis (PLS-DA) along with S-plot were generated using SIMCA, as shown in Fig. 1 which was reproduced and described from our previous study [1]. A heatmap was also generated using MetaboAnalyst to visualize the isoflavone distribution across the samples as shown in Fig. 2. Using the following criteria, the data was filtered for statistical analysis: VIP > 1.5, log_2_(FC) > 1, and *p* < 0.05, to identify potential discriminative metabolites associated with differences in antioxidant activity among the samples. In the study, a total of 33 metabolites were determined to be the most significant variable influencing differences between the samples based on our criteria as shown in Table 1 which was reproduced from our previous study [1]. To assess the significance of variables (features) in separating between classes or groups in MVDA, the metabolites were further visualized in an S-plot. Some metabolites were shown separated and contribute to the active samples of CTD and OTD. The dataset comprises raw data in mzML format and pre-processed list of metabolites mass spectra in excel format for both positive and negative ionisation that can be accessed for future metabolomics study.

**Table 1:** VIP values of 33 important differential metabolites in raw soybeans, in tempe after fermentation and in tempe digesta following *in vitro* digestion. This table was reproduced from Mio Asni et al. (2024), *Food Research International*, 195, 114951 [1].

No.	Metabolite name	VIP value
1.	Vaccenate	10.17
2.	Linolenyl alcohol	8.25
3.	Conjugated linoleic acid	7.42
4.	Linoleamide	6.45
5.	8, 11, 15-Octadecatrienoic acid	6.09
6.	11,13,15-Octadecatriensaure	5.58
7.	Daidzein	5.45
8.	Genistein	4.94
9	Alpha-linolenic acid	4.40
10.	Linoleic acid	4.40
11.	Dihydrodaidzein	3.95
12.	Biochanin A	3.88
13.	Monolinolenin	3.67
14.	Cholic acid	3.61
15.	Genistin	3.53
16.	Epoxyoleic acid	3.48
17.	Norvalin	3.15
18.	Daidzin	3.12
19.	Oleic acid	3.00
20.	4-Vinylphenol	2.97
21.	Linolenyl aldehyde	2.96
22.	9,12-Octadecadiynoic Acid	2.83
23.	*N*-(5-aminopentyl)octadec-9-enamide	2.80
24.	D-phenylalanine	2.57
25.	Farnesylacetol	2.42
26.	Prunetol	2.30
27.	Dimorphecolic acid	2.02
28.	Pidolic acid	2.01
29.	1-18:2-lysophosphophatidyl	2.00
30.	Indoline	1.94
31.	Glyceryl dilinoleate	1.93
32.	Glycitein	1.89
33.	Glutamate	1.85

## 4 Experimental Design, Materials and Methods

### 4.1 Sample preparation & extraction

**Fig. 3:**
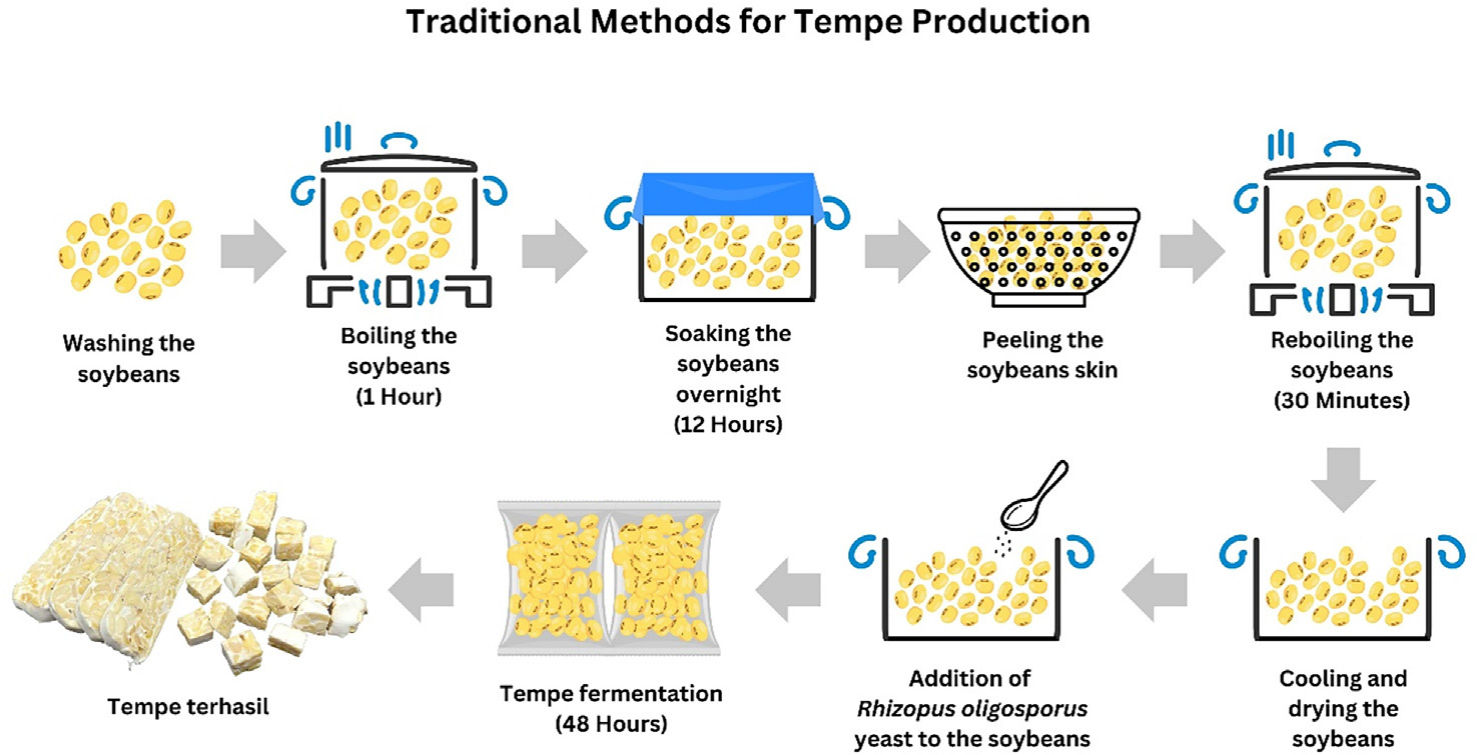
Traditional methods for tempeh production by local entrepreneurs.

Commercial organic and conventional tempe was produced by a local manufacturer, Perusahaan Tempe dan Tauge, situated in Sepang, Selangor. The tempeh samples were produced by a local manufacturer using traditional methods, as shown in Fig. 3. The inoculum used is *Rhizopus oligosporus* and the fermentation process is carried out for 48 h at a temperature of 27 °C and relative humidity of 75–85 %, reflecting the traditional tempeh fermentation conditions. Each sample were collected and subsequently cut into small cubes sized 2 cm³. Liquid nitrogen was added for 5 min to quenched each sample and the samples were freeze dried. The dried samples were then milled into coarse powder and stored at −20 °C [2]. Maceration method was used by soaking 500 g of sample powders in 700 mL of ethanol for 24 h. The ethanol solvent was subsequently evaporated from the samples using a rotary evaporator (50 °C, 90 mbar). Four replicates (n=4) were prepared from each sample group for each extraction, with blank digesta and blank ethanol as controls.

### 4.2 Tempe *in vitro* digestion in the gastrointestinal model

*In vitro* digestion was conducted following a modified static model to simulate oral, gastric, and intestinal phases [3]. Ground samples (1.5 g) of conventional and organic tempe were mixed with 5 mL of water to create a food paste. Simulated salivary fluid (SSF) was prepared by adding 4 mL of electrolyte solution, CaCl₂, and NaOH to adjust the pH to 7, followed by 0.25 mL of amylase. The mixture was incubated at 37 °C for 2 min. For the gastric phase, the pH was reduced to 3 with simulated gastric fluid (SGF), CaCl₂, and HCl, after which gastric lipase and pepsin were introduced. In the intestinal phase, the pH was raised to 7 by adding simulated intestinal fluid (SIF), bile salts, CaCl₂, and pancreatin, with incubation at 37 °C for 2 h. After digestion, samples were refrigerated overnight to halt enzyme activity, freeze-dried, and ground into powder for further analysis.

### 4.3 Ultra-high performance liquid chromatography–tandem mass spectrometry (UHPLC–MS/MS) analysis

Thermo Fisher Scientific's Ultimate 3000 Series system, which includes the WPS-3000RS autosampler, TCC-3000RS column compartments, and RS pump, was used to perform the analysis. Data processing is performed using Chromeleon 7.2 software, developed by Dionex Softron GMbH and Thermo Fisher Scientific in Waltham, MA. Data collection was carried out with slight modifications based on a study by Kasim et al. [4]. For separation, the ACQUITY UPLC® BEH C18 analytics column along with the Van Guard BEH C18 pre-column were maintained at 40 °C. The moving phase is delivered at a flow rate of 0.3 mL/min with an injection volume of 1 μL, consisting of a solution of A (0.1 % v/v formic acid in water) and B (0.1 % v/v formic acid in acetonitrile). HRMS data were obtained using the Thermo Scientific Q-Exactive Orbitrap mass spectrometer (Thermo Fisher Scientific, Waltham, MA), while analyte ionization was performed through critical thermal electrospray ionization (HESI). The scanning range of the centroid spectrum is set to range from 100–1500 m/z, with the five strongest peaks from each of the two ionization modalities selected for fragmentation. A total of four replicates (n = 4) of samples were randomly analyzed with solvent blank, pooled QC, and pooled external standards.

### 4.4 Advanced data processing with high-resolution mass spectrometry (HRMS)

ProteoWizard's MassConvert program was used to convert the MS raw data files into mzML format using. The HRMS data was then imported into MZmine version 3.7.2. The LCMS raw data was converted into a comprehensive list of features using the software. The list of features was then used for metabolite identification and statistical analysis. A published technique provided by Schmid et al. (2023) was applied as a protocol for LCMS data processing [5]. The feature list was then exported in two different formats, a summary file for spectra (.mgf) and a quantification table (.csv).

### 4.5 Computational annotation using GNPS and SIRIUS

High throughput dereplication of MS/MS data was produced using the Global Natural Products Social Molecular Networking (GNPS) platform [6]. The spectra in this network were then cross-referenced with the GNPS spectral libraries according to matching peaks between the experimental MS/MS spectrum and library spectra. MS2 spectral cosine similarity, with a cosine score threshold of at least 0.7 and six matching peaks were used as the parameter to annotate the unknown metabolites. The SIRIUS software version 5.8.2 was also used to annotate metabolite through molecular formula using orbitrap with MS2 mass accuracy of 5 ppm, possible ionization of [M + H]^+^, candidate molecular formulas of 3 and filtered by formulas from biological databases [7]. Then, fingerprint predictions were used to produce the CSI: FingerID module [8]. Feature-based molecular networking approach (FBMN) on the Global Natural Products Social Molecular Networking (GNPS) platform were used to produce molecular network (MN). Then, MN was built by setting filtering conditions for edges that have a cosine score above 0.7 and at least 6 corresponding peaks. Additionally, the network only maintains edges connecting two nodes if each node belongs to the top ten nodes with the highest level of similarity.

### 4.6 Multivariate data analysis using SIMCA

Multivariate data analysis (MVDA) was conducted according to the protocol set by Macintyre et al. [9]. The data in CSV format was imported into the SIMCA 14.0 software. A preliminary unsupervised statistical analysis such as Principal component analysis (PCA) was produced to determine the overall variance between soybean, tempe, and tempe digesta samples as the prediction variables and secondary metabolites as the responses. Partial Least Squares-Discriminant Analysis (PLS-DA) was used as the supervised analysis to investigate the effects of particular variables (metabolites) on cluster formation. Pareto scaling was employed in both PCA and PLS-DA to minimize the influence of large peaks while enhancing the visibility of smaller peaks that may hold biological significance. This scaling method reduces the dominance of highly abundant metabolites without distorting the overall data structure, allowing for a more balanced representation of metabolites with varying abundances. As a result, the multivariate analysis becomes more interpretable and meaningful, capturing relevant variations across both major and minor compounds. In PLS-DA, the value of the Variable Importance in Projection (VIP) index was used to assess the importance of a particular metabolite features in the spectra. A VIP scores > 1.5, log_2_ fold change, log_2_(FC) > 1, and significant difference, *p* < 0.05 was chosen as the criteria for metabolites with a high level of discrimination between classes. S-plot which depicted the covariance (*p*) against the correlation (*pcorr*) were then used to visualized the data. Heat maps of isoflavone concentration distributions across different samples were generated using MetaboAnalyst 5.0 software.

### 4.7 Statistical analysis

Each sample type was tested in four replicates for all experiment. The data was analysed using GraphPad Prism 9.0 (GraphPad Software, San Diego, CA, USA), and the results were expressed as mean ± standard deviation. One-way variance analysis (ANOVA) is used for statistical comparison, while the Tukey Post Hoc test was used for pair comparison analysis. A significant difference is indicated by a *p*-value of less than 0.05 (*p* < 0.05).

## Limitations

The study utilized UHPLC-HRMS/MS for the annotation of metabolites under various conditions of tempe samples. This approach yields just putative identification and does not provide quantitative concentration information.

## Ethics Statement

The authors have adhered to the ethical guidelines for publication in Data in Brief and confirm that the present work does not involve human subjects, animal experimentation, or data obtained from social media platforms.

## CRediT Author Statement

The data analysis was conceptualised and supervised by Nurkhalida Kamal, Norazlan Mohmad Misnan, Syarul Nataqain Baharum and Ahmed Mediani. Nurul Syahidah Mio-Asni carried out the formal analysis, investigation, data curation and visualization as well as writing the original manuscript. Data curation and validation were also assisted by Nurul Amalia Zahari and Ivana Nur Allisya. The paper was revised by Nurkhalida Kamal. All authors have read and approved the final published version of the work. All authors have read and agreed to the published version of the manuscript.

## Data Availability

Mendeley DataMass Spectrometry Dataset of Conventional and Organic Tempe Before and After In Vitro Digestion (Original data).

## Appendix Supplementary materials

Application 1
