# Supplementary material for: Mass spectrometry dataset of conventional and organic tempe before and after in vitro digestion
Source: Data Brief. 2025 Jun 23;61:111821. doi: 10.1016/j.dib.2025.111821 (PMC12266556; doi:10.1016/j.dib.2025.111821)
Supplement: Supplementary file 1 [file mmc1.pdf]

Food Research International 195 (2024) 114951

Contents lists available at ScienceDirect

Food Research International

journal homepage: www.elsevier.com/locate/foodres

Metabolomics insights of conventional and organic tempe during *in vitro* digestion and their antioxidant properties and cytotoxicity in HCT-116 cells

Nurul Syahidah Mio Asni [a], Reggie Surya [b], Norazlan Mohmad Misnan [c], Seng Joe Lim [d,e], Norzila Ismail [f], Shahrul Razid Sarbini [g], Nurkhalida Kamal [a,*]

[a] *Institute of Systems Biology (INBIOSIS), Universiti Kebangsaan Malaysia (UKM), Bangi 43600, Malaysia*
[b] *Food Technology Department, Faculty of Engineering, Bina Nusantara University, Jakarta 11480, Indonesia*
[c] *Herbal Medicine Research Centre, Institute for Medical Research, National Institutes of Health, Ministry of Health Malaysia, Shah Alam 40170, Selangor, Malaysia*
[d] *Department of Food Sciences, Faculty of Science and Technology, Universiti Kebangsaan Malaysia, UKM Bangi, Selangor 43600, Malaysia*
[e] *Innovation Centre for Confectionery Technology (MANIS), Faculty of Science and Technology, Universiti Kebangsaan Malaysia, UKM, Bangi, Selangor 43600, Malaysia*
[f] *Department of Pharmacology, School of Medical Sciences, Universiti Sains Malaysia, Kelantan, Malaysia*
[g] *Department of Crop Science, Faculty of Agricultural and Forestry Sciences, Universiti Putra Malaysia Kampus Bintulu Sarawak, Bintulu, Malaysia*

A R T I C L E I N F O

Keywords:
Metabolomics
Tempe
In vitro digestion

A B S T R A C T

Tempe, a fermented soybean food rich in polyphenols including isoflavones, is valued for its health benefits, notably its antioxidants. Concerns about glyphosate residues in crops have led to increased demand for organic soy products, including tempe. The study aimed to investigate the metabolomic profiles of tempe and its bioactive potentials prior to and following *in vitro* simulated gastrointestinal digestion. Conventional soybean (CS), conventional tempe (CT), conventional tempe digesta (CTD), organic soybean (OS), organic tempe (OT) and organic tempe digesta (OTD) were analysed for various assays. The study observed a significant decrease in the total phenolic and flavonoid levels for conventional and organic samples in tempe extracts (CT, OT) compared to tempe digesta (CTD, OTD). Organic tempe digesta has a higher total phenolic content (CTD = 22.55 µg GAE/g, OTD = 41.36 µg GAE/g) and flavonoid content (CTD = 4.64 µg QE/g, OTD = 10.06 µg QE/g) compared to conventional tempe digesta. However, there is a significant difference in the bioaccessibility of phenolic (CT = 74.77 %, OT = 59.20 %) and flavonoid (CT = 49.4 %, OT = 57.52 %) in both organic and conventional tempe. Tempe consistently surpasses soybean in antioxidant assays such as DPPH, ABTS, and FRAP. Organic tempe digesta exhibits the most elevated levels of antioxidants. Using GNPS and the SIRIUS database, 34 metabolites were annotated according to the criteria of having a VIP score > 1.5, a log2(FC) > 1, and a p-value < 0.05. From the list, 26 metabolites demonstrated a positive correlation with antioxidant activity, DPPH, and FRAP. Molecular networking enables the visualization of 12 prominent isoflavones, namely daidzein, daidzin, genistein, genistin, glycitein, glycitin, 6″-O-malonyldaidzin, 6″-O-acetylgenistin, 6″-O-acetyldaidzin, and 7,8,4′-trihydroxyisoflavone. Interestingly, aglycone isoflavones are abundant in organic tempe digesta while glycoside isoflavones are abundant in organic and conventional soybeans. Overall, the findings indicate that tempe digesta exhibits distinct metabolic patterns and bioactive potentials.

1. Introduction

Soybeans are commonly grown with glyphosate, an herbicide that can disrupt plant growth and protein production, with residues linked to various health risks (Kanissery et al., 2019; Meftaul et al., 2020). While opting for organic soybeans can reduce these risks, studies have also shown that organic vegetables contain higher phenolic content and better nutritional profiles (Yu et al., 2018). This has increased the demand for organic soybean products including tempe.

Tempe is a traditional Indonesian fermented soybean food, rich in protein, vitamins, and minerals (Wu & Hasnah, 2018). It is made by soaking, dehulling, and boiling soybeans, which are then ferment with *Rhizopus* sp., resulting in a firm, cake-like product. Tempe offers numerous health benefits, including high antioxidant levels and

* Corresponding author.
E-mail address: nurkhalida.kamal@ukm.edu.my (N. Kamal).

https://doi.org/10.1016/j.foodres.2024.114951
Received 7 April 2024; Received in revised form 16 August 2024; Accepted 20 August 2024
Available online 23 August 2024
0963-9969/© 2024 Elsevier Ltd. All rights reserved, including those for text and data mining, AI training, and similar technologies.

potential cancer prevention due to the presence of its secondary metabolites (Astuti et al., 2000). For instance, it has isoflavones like genistein, daidzein, glycitein, and factor-2 (6,7,4′-trihydroxyisoflavone), which are antioxidants that can inhibit the formation of cancer-causing free radicals (Klus, Burger-Papendorf, & Barz, 1993). Polyunsaturated fatty acids (PUFAs) are also responsible for the bioactivity of tempeh, as soybeans are abundant in linoleic, oleic, and linolenic acids (Limanjaya, Subali, & Yanti, 2022).

In vitro static digestion systems simulate the digestive process, breaking down food into bioactive metabolites that can exhibit anti-inflammatory, antioxidant, and anticancer effects (Soumya et al., 2021). Metabolomics analysis can be used to observe the changes in metabolite composition during different digestion stages, including the oral, gastric, and intestinal phases. Through techniques like liquid chromatography-mass spectrometry (LCMS), these metabolites can be analysed to investigate their breakdown and release during digestion (Rocchetti et al., 2020; Vazquez-Aguilar et al., 2023).

Current research reveals no significant metabolite profile variations between conventional and organic tempe (Chong et al., 2023). However, there is a lack of studies on their metabolite profiles post-digestion and their cytotoxicity effects. This study aims to evaluate the changes in metabolites after *in vitro* digestion and determine their antioxidant capacity and cytotoxic potential on HCT-116 cells, highlighting the potential benefits of organic tempe as a source of bioactive metabolites.

2. Material and methods

2.1. Chemicals and reagents

Conventional soybeans were obtained from a local producer, whereas organic soybeans with organic certification were purchased online from Justlife®. Tempe inoculum RAPRIMA was used as a starter (PT Aneka Fermentasi Industri, Bandung, Jawa Barat, Indonesia). The cell culturing and cytotoxicity experiments of human liver normal cell lines, WRL-68 and human adenocarcinoma cell lines, HCT-116 were conducted using Dulbecco's Modified Eagle Medium (DMEM) and Minimum Essential Medium (MEM) obtained from Bio-Diagnostics Sdn. Bhd. The solvents utilised for both sample preparation and mass spectrometry analysis were of LCMS quality, and they were acquired from Chemiz.

2.2. Sample preparation

Commercial organic and conventional tempe was prepared by a local producer, *Perusahaan Tempe dan Tauge* located in Sepang, Selangor. All samples were obtained on the same day and samples were cut into a small cube (2 cm^3). Each sample was quenched with liquid nitrogen for 5 min to stop the microbial activity and freeze dried to remove moisture. The dried samples were then milled into coarse powder and stored at −20 °C (Nowak & Jakubczyk, 2020).

2.3. Extraction

The maceration method was used to extract conventional soybean (CS), conventional tempe (CT), and conventional tempe digesta (CTD), along with organic soybean (OS), organic tempe (OT), and organic tempe digesta (OTD). The sample powders (500 g) were soaked in 700 mL of ethanol for 24 h. Following the extraction, the ethanol solvent was eliminated from the samples utilising a rotary evaporator (50 °C, 90 mbar). For every extraction, four replicates (n = 4) of each sample group including blank digesta and blank ethanol as controls were prepared.

2.4. Simulated gastrointestinal (GI) digestion of tempe

Based on the technique described by Mulet-Cabero et al. (2020), a static model was adapted to simulate oral, gastric and intestinal human digestion. Approximately 1.5 g ground conventional and organic tempe powder was diluted in 5 mL of water to simulate the solid food paste. Electrolyte stocks were then prepared according to the concentration determined in the previous method (Mulet-Cabero et al., 2020). The stocks were kept and pre-warmed in a shaking incubator at 37 °C. At the beginning of the oral phase, a total of 4 mL of electrolyte simulated salivary fluid (eSSF) solution was added to the paste sample and the pH of the sample was measured. Then, 0.03 mL $CaCl_2(H_2O)_2$ was added to the mixture. Approximately 0.03 mL of 1 mol/L NaOH was added to the sample until pH 7 is obtained simulating the neutral oral phase environment and 0.97 mL of water was added to obtain the required concentration of simulated salivary fluid (SSF). A total of 0.25 mL amylase was then added to the SSF. The time of contact with amylase in the shaking incubator is 2 min at 37 °C. The pH was then decreased from neutral pH of 7 to pH 3 in gastric phase by adding the 10 % of electrolyte simulated gastric phase (eSGF) volume (0.7 mL) to the SSF solutions from the oral phase, which was priorly adjusted using 1 mol/L HCl to the pH of 2. The mixture of the remaining 6.3 mL eSGF, 0.0043 ml $CaCl_2(H_2O)_2$, and the required amount of water (1.6 ml) without enzymes were added gradually to make up the other 90 % of the simulated stomach electrolyte mixture. Approximately 0.4 mL of 1 mol/L HCl was added to ensure that it reached pH 3 by the end of the gastric digestion. The mixture was mixed with 1 mL of gastric lipase and 1 mL of pepsin. The pH was increased from pH 3 in the gastric phase to pH 7 following the neutral pH in the intestine which is achieved by adding 12.5 mL of electrolyte simulated intestinal phase (eSIF) solution with pH 7 to the simulated gastric fluid (SGF) solution from the gastric phase. The mixture was then added with 2.5 mL of bile salts, followed by 0.04 mL of $CaCl_2(H_2O)_2$. Then, 1 mL of pancreatin was introduced to the mixture. The mixture was added with 3.81 mL of water to achieve the required concentration of simulated intestinal fluid (SIF). At 37 °C, the intestinal digestion phase was held for two hours in a shaking incubator. After all the phases are finished, the samples were put to rest for 15 min before stored in the fridge for one day to stop all the enzyme activity. A final ratio of product from the prior phase to the next phase is 1:2 (*v/v*). The resulting liquid digesta for both conventional and organic were then freeze dried and ground into powdered digesta samples.

2.5. Total phenolic and flavonoid contents (TPC & TFC)

The microplate total phenolic content (TPC) method utilised in this study employed a modified version of the 96-well microplate Folin–Ciocalteu method developed by Bristy et al., (2022). Standard solutions of gallic acid were prepared at concentrations of 25, 50, 100, 150, and 200 μg/ml diluted in ethanol. Sample extracts were prepared by diluting 1 mg samples with 1 ml of 100 % ethanol. A mixture of 30 μL from 1 mg/ml of the ethanolic sample extract and gallic acid standard was combined with 120 μL of a 10 % (*v/v*) Folin-Ciocalteau reagent solution, followed by the addition of 150 μL of a sodium carbonate solution (7.5 % *w/v*). Following a 2-hour incubation of the mixture, the absorbance at 765 nm was measured. The results were measured in micrograms of gallic acid equivalents (GAE) per gram of the extract.

The total flavonoid content (TFC) was determined using a method from Sembiring et al. (2018) with aluminium chloride method. Standard solutions of quercetin were prepared with concentrations of 20, 40, 60, 80, and 100 μg/ml diluted in ethanol. The well was added with 50 μl of sample ethanolic extracts (1 mg/ml) or the ethanolic standard solution, followed by 10 μl of a 10 % aluminium chloride solution and 150 μl of ethanol. Subsequently, 10 μl of 1 M sodium acetate was introduced to the mixture in a 96-well plate. A reagent blank of ethanol served as a control. After thorough mixing, the solution was shielded from light and incubated for 40 min at room temperature. Microplate reader was used to measure the absorbance at 415 nm. The quantification of flavonoids was measured in micrograms of quercetin equivalents (QE) per gram of sample extract.

2.6. Bioaccessibility index

The bioaccessibility index indicates the quantity of phenolic and flavonoid content that are released after gastrointestinal digestion and potentially available for absorption (Santana Andrade et al., 2022). The index was measured using the following formula:

$$\text{Bioaccessibility index } (\%) = \frac{A}{B} \times 100$$

where,
A = TPC/TFC following *in vitro* digestion
B = TPC/TFC prior to *in vitro* digestion

2.7. Antioxidant activities

2.7.1. Free radical scavenging activity assay (DPPH)

The scavenging activity of tempe extracts was evaluated by the presence of DPPH radicals according to the method reported by Athilah et al. (2020). 1 mg samples and standards were dissolved in 1 ml of 100 % ethanol to prepare a stock solution with a concentration of 1 mg/mL. The solution was further diluted to varying concentrations (15.63, 31.25, 62.50, 125, 250, 500, and 1000 µg/mL). A control blank solution containing ethanol and DPPH was also prepared. The samples (50 µL) were well mixed with 150 µL of a 0.1 mM DPPH working solution on 96-well plates. The solution was then left in darkness for 30 min. Trolox was used as a standard. Absorbance at 515 nm was determined using a microplate reader. The following equation was used to calculate the percentage of DPPH scavenging activity based on results of 1000 µg/mL concentrations:

$$Freeradicalscavengingactivity(\%) = [(A0 - A1)/A0] \times 100$$

(A_0 = absorbance of control, A_1 = absorbance of sample).

2.7.2. Ferric reducing antioxidant power assay (FRAP)

The FRAP assay was conducted using the methodology described by Athilah et al. (2020). Sample extracts and standards were initially dissolved in ethanol to create a stock solution with a concentration of 1 mg/mL. From this stock solution, other concentrations (15.63, 31.25, 62.50, 125, 250, 500, and 1000 µg/mL) were created by further dilution. A concentration of 300 mM acetate buffer was made by combining 0.16 g of sodium acetate with 100 mL of 0.28 M glacial acetic acid in pH 3.6. A 10 mM solution of 2,4,6-tripyridyl-s-triazine was made by dissolving 0.31 g of 2.4.6-tripyridyl-s-triazine in 100 mL of 40 mM hydrochloric acid (HCl). FRAP reagent solution was produced by mixing 10 mL of acetate buffer with a pH of 3.6, 1 mL of 2,4,6-tripyridyl-s-triazine in hydrochloric acid (40 mM), and 1 mL of ferric chloride (20 mM), maintaining a 10:1:1 ratio. The sample extract (50 µL) was combined with 0.15 mL of the FRAP reagent. The mixture was then kept in the dark for 30 min. The absorbance was determined at a wavelength of 593 nm, and the percentage of inhibition was measured for 1000 µg/mL concentrations using the equation, where A_1 represents the sample's absorbance and A_0 denotes the control's absorbance.

$$Inhibition(\%) = [(A1 - A0)/A1] \times 100$$

(A_0 = absorbance of control, A_1 = absorbance of sample).

2.7.3. 2,2′-azino-bis(3-ethylbenzothiazoline-6-sulfonic acid) radical cation-based assay (ABTS)

ABTS (2,2′-azinobis-3-ethylbenzothiazoline-6-sulphonic acid) radical-scavenging activity was assessed using a slightly modified version of the methodology by Morales & Paredes (2014). A 7 mM ABTS solution and 2.45 mM potassium persulfate were combined to create ABTS radical cations, which were then allowed to sit at room temperature in the dark for a duration of 12–16 h. After measuring the absorbance of the resulting solution at 734 nm, ethanol was used to adjust the absorbance to 0.7. Sample extracts and standards were prepared with 100 % ethanol. In a 96-well microplate, 20 µL from 1 mg/ml of various concentration of tempe extracts and standards (15.63, 31.25, 62.50, 125, 250, 500, and 1000 µg/mL) was combined with 180 µL of the ABTS reagent, and after a 6 min period, the absorbance at 734 nm was measured using a microplate reader. Trolox was used as positive controls. The ABTS scavenging activity percentage was measured for 1000 µg/mL concentrations according to the equation where, A_0 = absorbance of the control and A_1 = absorbance of the sample.

$$Freeradicalscavengingactivity(\%) = [(A0 - A1)/A0] \times 100$$

(A_0 = absorbance of control, A_1 = absorbance of sample).

2.8. Cytotoxicity against HCT-116 cell lines

In order to assess the cytotoxicity of tempe extracts, a 3-[4,5-dimethyl thiazol-2-yl] 2,5-diphenyl tetrazolium bromide (MTT) assay was performed, following the method outlined by Faliq et al. (2020). Colon adenocarcinoma cells (HCT-116) and normal liver cells (WRL-68) were cultured in 96-well flat-bottomed plates. Each well contained 5×10^4 cells in a volume of 100 µl/well of DMEM and MEM medium, respectively. After incubating for 24 h at 37 °C with 5 % CO_2, tempe extracts were prepared using 100 % ethanol with concentration ranging from 3.9 µg/ml to 1000 µg/ml were applied to the cells. A mixture of sample solvents (80 % DMSO with 20 % media) was used as the negative control, while Fluorouracil was used as the positive control at concentrations ranging from 0.6 to 198 µg/ml. Following a 72-hour period of incubation, each well was added with 30 µl of MTT solution (2 µg/ml), and the plates were then incubated at 37 °C for 4 h. Subsequently, 200 µl of DMSO was used to dissolve the MTT crystals, and the absorbance at 570 nm was measured. The absorption values at this wavelength directly represented the relative cell populations compared to the control group. Each concentration was added into four wells, and each extract was tested for two times (n = 2). The IC_{50} values for both treated cancer and normal cells were determined using the provided formula to assess the cytotoxic effects of all extracts.

$$Percentageofcellviability(\%) = \frac{Absorbanceoftreatedcells}{Absorbanceofcontrol} \times 100$$

2.9. Ultra-high performance liquid chromatography-tandem mass spectrometry (UHPLC-MS/MS) analysis

The analysis was conducted using the Thermo Fisher Scientific Ultimate 3000 Series WPS-3000RS autosampler, Thermo Scientific Dionex Ultimate 3000 Series TCC-3000RS column compartments, and Thermo Scientific Dionex Ultimate 3000 Series RS pump. The Chromeleon 7.2 software, developed by Thermo Fisher Scientific in Waltham, MA and Dionex Softron GMbH (a part of Thermo Fisher Scientific in Germany), was used for data processing. Data collection was conducted as described by Kasim et al. (2023) with some modification. The separations were conducted using an ACQUITY UPLC® BEH C18 analytical column (2.1 mm × 100 mm; particle size, 1.7 µm) (Waters, Milford, MA, USA) with a Van Guard BEH C18 pre-column (2.1 mm × 5 mm; particle size, 1.7 m) (Waters, Milford, MA, USA) kept at a temperature of 40 °C. The mobile phase comprised of solutions A (0.1 %v/v formic acid in water) and B (0.1 % v/v formic acid in acetonitrile solution). The elution process involved a gradient program, starting with a 5 % solution B for 2 min followed by a gradual increase from 5 % to 100 % solution B between 3.0 and 17.0 min. From 17.0 to 19.0 min, a 100 % solution B was utilized, and then the concentration was reduced back to 5 % solution B between 19.5 and 24.0 min. The mobile phase was delivered at a flow rate of 0.3 mL per min with an injection volume of 1 µL. The instrument operated at a resolution of 70,000 in full scan mode and 35,000 in MS/MS scan mode. The critical heat electrospray ionization (HESI) source parameters were fine-tuned with the following settings: a spray voltage

of 4.0 kV, capillary temperature set at 320 °C, sheath gas flow rate at 35, auxiliary gas flow rate at 102, heater temperature at 350 °C, and an S-lens RF level of 55. The spectrum data type was centroid with a scan range from 100 to 1500 *m/z*. The maximum permissible accumulation time (IT) was 60 ms, and the target value (AGC) was 1 × 105. A TopN = 5 method, MS/MS (ddMS2) analyses were used for the data-dependent. In both ionisation modalities, five strongest peaks were chosen for fragmentation at stepped normalised energy of 15, 30, and 35 V. The samples in four replicates (n = 4) were run randomly together with solvent blank, pooled QC and pooled external standards.

2.10. High-resolution mass spectrometry (HRMS) data processing

The raw data files of MS were transformed into the format of mzML through the MassConvert tool in ProteoWizard. The acquired HRMS data were then imported into Mzmine software version 3.7.2. This software is employed to process the LCMS raw data into a comprehensive list of features that can be utilised for further downstream analyses including metabolite identification and statistical analysis. The LCMS data processing workflow was adapted from a published protocol outlined by Schmid et al. (2023). Initially, the raw data processing step using the mass detection module was done to generate a mass list encompassing *m/z* values surpassing the designated noise threshold for both MS level 1 and 2. Feature processing was executed, involving the chromatogram builder, chromatogram deconvolution, and isotope removal, thereby obtaining a list of all identified features from the LCMS data. Then, feature alignment and gap-filling steps were carried out. The resulting feature list was subsequently exported in two distinctive formats: a quantification table (.csv) and a summary file for spectra (.mgf).

2.11. Molecular network analysis and computational annotation using GNPS and SIRIUS

The spectra within this network were cross-referenced with the GNPS spectral libraries The Global Natural Products Social Molecular Networking (GNPS) platform was utilised to produce high throughput dereplication of MS/MS data (Wang et al., 2016). This process involved querying the obtained MS/MS spectral data against the extensive library of MS/MS spectra within the GNPS spectral libraries. The annotation of unknown metabolites was conducted based on the MS2 spectral cosine similarity, with a cosine score threshold of above 0.7 and a minimum of 6 matched peaks.

Metabolite annotation was performed using SIRIUS software version 5.8.2, which incorporated the identification of molecular formula and the determination of possible molecular fingerprints based on the fragmentation tree and unknown compounds spectrum (Dührkop et al., 2019). The parameters used for SIRIUS molecular formula identification were as follows: instrument, orbitrap; MS2 mass accuracy, 5 ppm; possible ionisation, $[M+H]^+$; candidate molecular formulas, 3; filtered by formulas from biological databases. CSI: FingerID module was selected for fingerprint prediction using the following parameters: possible adducts: $[M+H]^+$, filter: compounds found in biological database (Rutz et al., 2019).

The Global Natural Products Social Molecular Networking (GNPS) platform, available at https://gnps.ucsd.edu, was used to create the molecular network (MN) using the feature-based molecular networking process (FBMN). Subsequently, a particular MN was created by implementing filtering conditions for edges, which required a cosine score exceeding 0.7 and a minimum of six matching peaks. Furthermore, the network only retained edges connecting two nodes if each node was in the top 10 nodes with the highest similarity to the other.

2.12. Multivariate data analysis

The multivariate data analysis (MVDA) was conducted according to the published protocol by Macintyre et al. (2014). The data in the CSV file was transferred into SIMCA 14.0. A unique primary ID was created in SIMCA and retention time (Rt), *m/z*, and molecular weight (MW) were regarded as secondary IDs. The data underwent a preliminary unsupervised statistical analysis employing principal component analysis (PCA) to observe the overall variance between the soybeans, tempe and tempe digesta samples (predictor variables) and the secondary metabolites (responses) produced from LCMS data. To assess the influence of specific variables (metabolites) on the clusters, supervised analyses like Partial Least Squares-Discriminant Analysis (PLS-DA) and Orthogonal Projection to Latent Structures Discriminant Analysis (OPLS-DA) were conducted. The OPLS-DA analysis was used to statistically discriminate and compare the most distinct sample with the other sample. PLS regression was employed to examine the notable variances among metabolites generated by three distinct sample groups (soybeans, tempe, and tempe digesta) and their corresponding bioactivities, including TPC, TFC, and antioxidant assays. Pareto scaling was implemented in PCA, PLS-DA, OPLS-DA, and PLS regression to minimise the impact of strong peaks while highlighting weaker peaks that may hold greater biological significance and model validity was assessed using multiple correlation coefficients (R^2), cross-validation (Q^2), and permutation tests for the supervised method. Heatmaps together with hierarchical clustering techniques (HCA) were generated using MetaboAnalyst software. The Variable Importance in Projection (VIP) index values were employed within an OPLS-DA to determine the significance of individual metabolite features within the spectra. Metabolites with VIP scores VIP > 1.5, log2 fold change log2(FC) > 1, and significant difference, $p < 0.05$ exhibit a high level of discriminatory information between classes.

2.13. Statistical analysis

All tests were performed with four replicates of each type of sample. The data was analysed using Graph Pad Prism 9.0 (Graph Pad Software, San Diego, CA, USA) and was then presented as mean value ± standard deviation. Analysis of variance one-way (ANOVA) is used for statistical comparisons, while the Tukey Post Hoc test is used to analyse pairwise comparisons. Significant differences are defined as having a p-value of less than 0.05 ($p < 0.05$).

3. Results

3.1. Total phenolic and flavonoid content

In this study, TPC and TFC in eight sample groups were determined and shown in Table 1. Overall, there is a significant difference in TPC of all sample extracts. The result shows that there is a statistically significant decrease in TPC and TFC between soybean for both conventional and organic products. Additionally, there is also a significant decrease in TPC between tempe and tempe digesta extracts. Meanwhile, the highest contain of flavonoids was shown present in organic soybean and the lowest was in conventional tempe digesta There is a statistically

Table 1
TPC and TFC of all samples (mean ± standard deviation). (A) TPC. (B) TFC. Different letters in superscript demonstrate statistically significant at $p < 0.05$ (One-Way ANOVA, Tukey Post-hoc test).

Samples	Total Phenolic Content (μg GAE/g)	Total Flavonoid Content (μg QE/g)
Conventional Soybean (CS)	48.60 ± 0.99[c]	14.78 ± 1.00[c]
Organic Soybeans (OS)	91.96 ± 4.56[a]	38.38 ± 0.55[a]
Conventional Tempe (CT)	30.16 ± 0.79[e]	9.39 ± 1.30[e]
Organic Tempe (OT)	69.87 ± 2.49[b]	17.49 ± 0.48[b]
Conventional Tempe Digesta (CTD)	22.55 ± 0.73[f]	4.64 ± 0.57[f]
Organic Tempe Digesta (OTD)	41.36 ± 2.52[d]	10.06 ± 0.47[d]

significant decrease in TFC from raw soybeans to tempe for both conventional and organic product. There is also a decrease in TFC for both conventional and organic tempe from before and after *in vitro* gastrointestinal digestion.

3.2. Bioaccessibility index

Bioaccessibility index was evaluated to determine the percentage of certain active compounds which are absorbed by the circulatory system. Table 2 shows the bioaccessibility index of phenolic and flavonoid content in conventional tempe was 13.40 ± 1.77 % and 8.57 ± 1.50 % and in organic tempe was 13.40 ± 1.77 % and 8.57 ± 1.50 %. There is a significant difference in the bioaccessibility of phenolic between organic tempe and conventional tempe. However, no significant difference was shown between the bioaccessibility of flavonoid for organic tempe and conventional tempe.

3.3. Antioxidant activity

Table 3 showed the findings for antioxidant assays including DPPH and ABTS scavenging activity as well as FRAP. The findings revealed that there are no significant changes of DPPH scavenging activity between soybeans for both organic and non-organic samples. However, another significant observation found from the result is the high antioxidant activity in tempe digesta for both non-organic and organic samples. The antioxidant activity is markedly higher than that of the undigested tempe sample. Meanwhile, the findings also revealed that organic samples showed significantly higher antioxidant activity than the non-organic samples for soybean and tempe.

The ABTS scavenging activity in Table 3 also revealed that tempe extract exhibited significantly higher scavenging activity than soybean. Similar findings can also be observed in tempe digesta sample, where they showed increased scavenging activity in comparison to tempe sample. Meanwhile, to evaluate organic and conventional samples, statistical findings demonstrated that there is no significant difference between conventional soybean and organic soybean as well as conventional tempe and organic tempe. However, a significant increase of scavenging activity can be observed between conventional tempe digesta and organic tempe digesta.

FRAP assay evaluates the antioxidant's capacity to reduce a ferric tripyridyltriazine (Fe^{3+}-TPTZ) complex, generating ferrous tripyridyltriazine (Fe^{2+}-TPTZ) with colour. This reduction process occurs as compounds donate its hydrogen atom or electron, breaking the chains of free radicals (Mohamed Idris et al., 2015). The findings demonstrate a significant difference in inhibition percentage between soybean and tempe. A high significant finding in antioxidant activity can be observed in the digested tempe for the organic sample. This increase is substantial compared to the undigested tempe. Furthermore, a comparison between organic and non-organic samples revealed that organic samples consistently exhibit significantly higher antioxidant activity for each type than non-organic samples.

Table 2
Bioaccessibility index of phenolic and flavonoid content in conventional and organic tempe (%) after *in vitro* gastrointestinal digestion simulation. Different letters in superscript demonstrate statistically significant at $p < 0.05$ (One-Way ANOVA, Tukey Post-hoc test).

Digested Tempe Samples	Bioaccessibility of Phenolic (%)	Bioaccessibility of Flavonoid (%)
Conventional Tempe (CT)	74.77 ± 2.18^{a}	49.4 ± 3.20^{a}
Organic Tempe (OT)	59.20 ± 1.42^{b}	57.52 ± 2.11^{a}

Table 3
Percentage of scavenging & inhibition activity for antioxidant capacity assays (mean ± standard deviation). **(A)** Percentage of DPPH scavenging activity. **(B)** Percentage of ABTS scavenging activity. Trolox as a positive control. **(C)** Percentage of FRAP inhibition activity. Different letters in superscript demonstrate statistically significant at $p < 0.05$ (One-Way ANOVA, Tukey Post-hoc test).

Samples	DPPH (%)	ABTS (%)	FRAP (%)
Conventional Soybean (CS)	12.12 ± 3.16^{cd}	19.22 ± 2.16^{d}	25.15 ± 0.42^{d}
Organic Soybeans (OS)	22.04 ± 2.65^{c}	23.53 ± 3.31^{d}	34.80 ± 22.55^{c}
Conventional Tempe (CT)	13.83 ± 1.39^{d}	31.46 ± 2.78^{cd}	31.12 ± 0.69^{c}
Organic Tempe (OT)	22.24 ± 1.13^{cd}	41.79 ± 2.00^{c}	48.06 ± 0.511^{b}
Conventional Tempe Digesta (CTD)	39.88 ± 1.18^{b}	49.45 ± 0.92^{b}	28.75 ± 2.52^{cd}
Organic Tempe Digesta (OTD)	48.40 ± 1.24^{a}	69.15 ± 4.36^{a}	56.86 ± 2.12^{a}

3.4. Cytotoxicity against cancer cell lines

Cytotoxicity activity of sample groups was determined on HCT116 and WRL68. The cytotoxicity activity of sample group extracts was determined using MTT assay and a graph of concentration of treatments (μg/ml) against cell viability (%) was plotted to determine the IC_{50} value and compare the percentage of cell viability across the treatments. Fig. 1A shows the cytotoxicity activity of sample group extracts against WRL68. All sample group extracts showed $IC_{50} > 500$ μg/ml which shows that all sample groups are not toxic to the WRL68. Additionally, organic soybean, tempe and digesta was shown less cytotoxic to normal cells compared to conventional soybean, tempe and digesta as the cell viability was shown higher for each treatment concentration. Fig. 1B shows the cytotoxicity activity of sample group extracts against HCT116. The result shows that only Fluorouracil exhibited moderate cytotoxicity with IC_{50} value < 50 between 125 and ^{10}00 μg/ml. All the sample group extracts showed no significant difference and did not exhibit cytotoxicity against the colon cancer cells HCT116. The lowest percentage of cell viability shown among treatment was organic tempe digesta with 52.44 % at 500 μg/ml.

3.5. Comparative metabolite changes between organic vs conventional samples of raw soybean, tempe and tempe digesta

The metabolite content of soybean, tempe, and tempe digesta was analyzed using LCMS, detecting 82 metabolites and 230 unidentified peaks. The prominent categories of discovered metabolites included organic acids, amino acids, sugars, glycosides, triterpenoids, polyacetylenes, phenylpropanoids and flavonoids. The variation in metabolite contents of all sample groups were evaluated through MVDA. PCA was utilized to elucidate the clustering characteristics exhibited by each sample and to discern the metabolites responsible for the observed variability. The PCA score plot illustrates the grouping of the samples, while the loading plot provides insight into the contributions of variables to the observed differences among the samples in the score plots. All samples within the score plots fall within the bounds of the 95 % Hotelling T2 ellipse. The LCMS data was processed and analyzed using PCA, and the findings are displayed in Fig. 2. The PCA score plot showed that the first principal component (PC1) accounted for 48.4 % of the variation, while the second component (PC2) explained 15.3 %. The score plot (Fig. 2A) revealed that the different stages of tempe were well separated into three clusters with distinctive difference between tempe digesta samples (CTD, OTD) to raw soybean (CS, OS) and tempe samples (CT, OT) at PC1. Conventional and organic soybeans were clustered together, having negative PC1 and positive PC2 scores respectively, indicating similar metabolite profiles, consistent with previous findings showing no significant differences between organic and non-organic

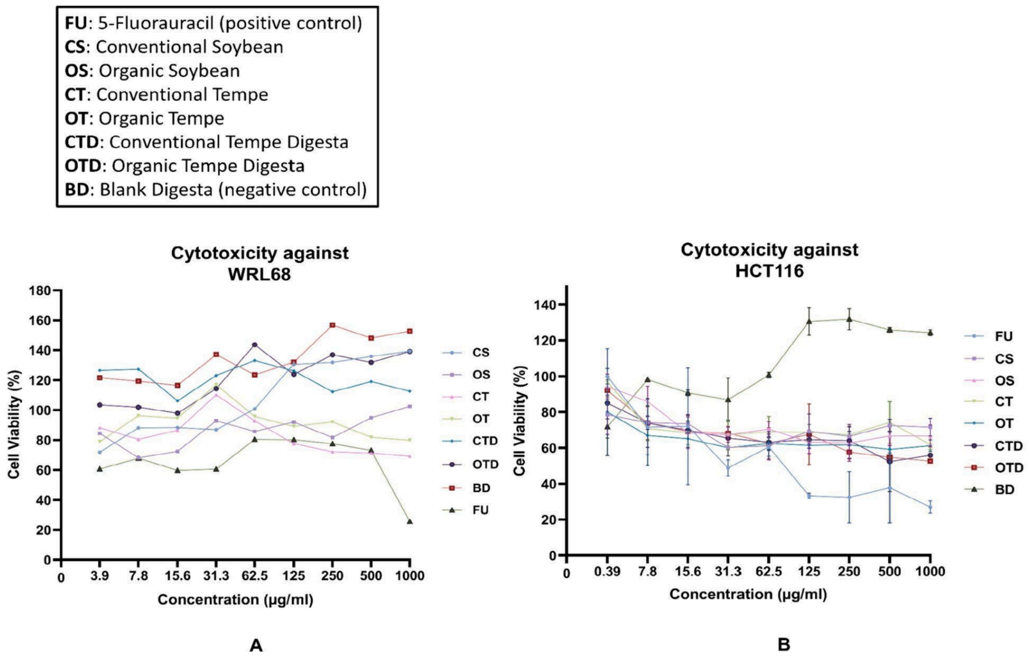

Fig. 1. Cytotoxicity activity of sample treatments against cell lines (mean ± standard deviation). **(A)** Cytotoxicity against human liver normal cell lines, WRL68. **(B)** Cytotoxicity against human colon cancer cell lines, HCT116.

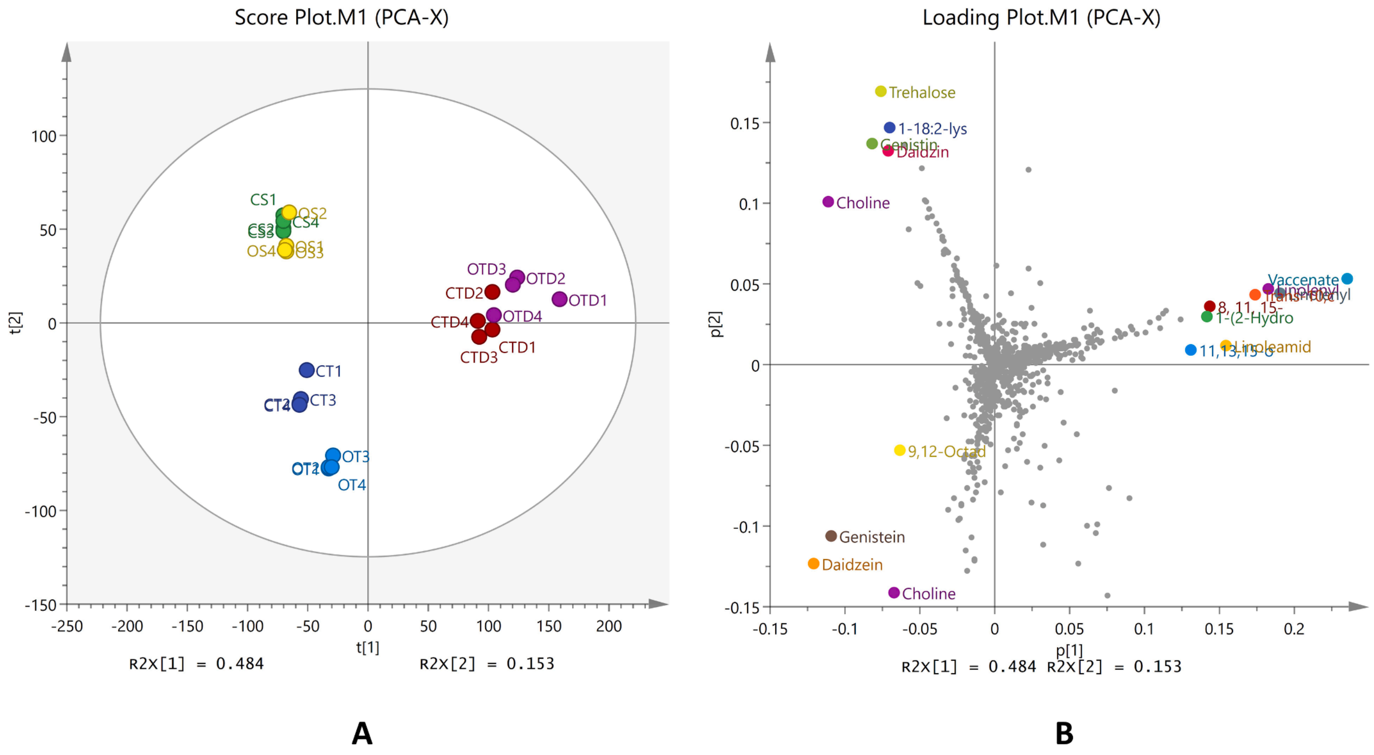

Fig. 2. The MVDA analysis for the normalised data was conducted using SIMCA software version 14.0. **(A)** PCA score plot derived from the LCMS profiles of different sample groups. **(B)** PCA loading plot showing the clustering of metabolites content in different sample groups.

soybeans (Chong et al., 2023). The loading plot (Fig. 2B) showed that the metabolites contribute to the separation of each sample group with each respective *m/z* value.

The loading plot (Fig. 2B) showed that the metabolites causing the separation of tempe digesta extracts from soybean and tempe extracts were mostly fatty acids including linolenyl aldehyde, linolenyl alcohol, 11,13,15-octadecatriensaure, 8,11,15-octadecatrienoic acid, linoleamide, *trans*-10,*cis*-12 conjugated linoleic acid, vaccenate and 1-(2-hydroxyethyl)- 2-(heptadecenyl)imidazole. Meanwhile, the tempe extract was separated from the other groups by having higher contents of daidzein, genistein, choline, and 9,12-octadecadienoic acid. Trehalose, daidzin, genistin and 1–18:2-lysophosphatidylcholine were found to be responsible for the separation of the soybean extract from the rest of the groups. Fig. S1 displays the initial pairing of individual calves

within their respective sample groups based on HCA. Subsequent progression paired individuals into three different kinds of samples: tempe, soybean, and tempe digesta. From the clustering, conventional and organic tempe digesta are clustered first in their own subgroups, showing high similarity within them compared to other groups. This is followed by conventional and organic soybeans which were also clustered in separate subgroups from conventional and organic tempe. The final clustering produces clear separation between conventional and

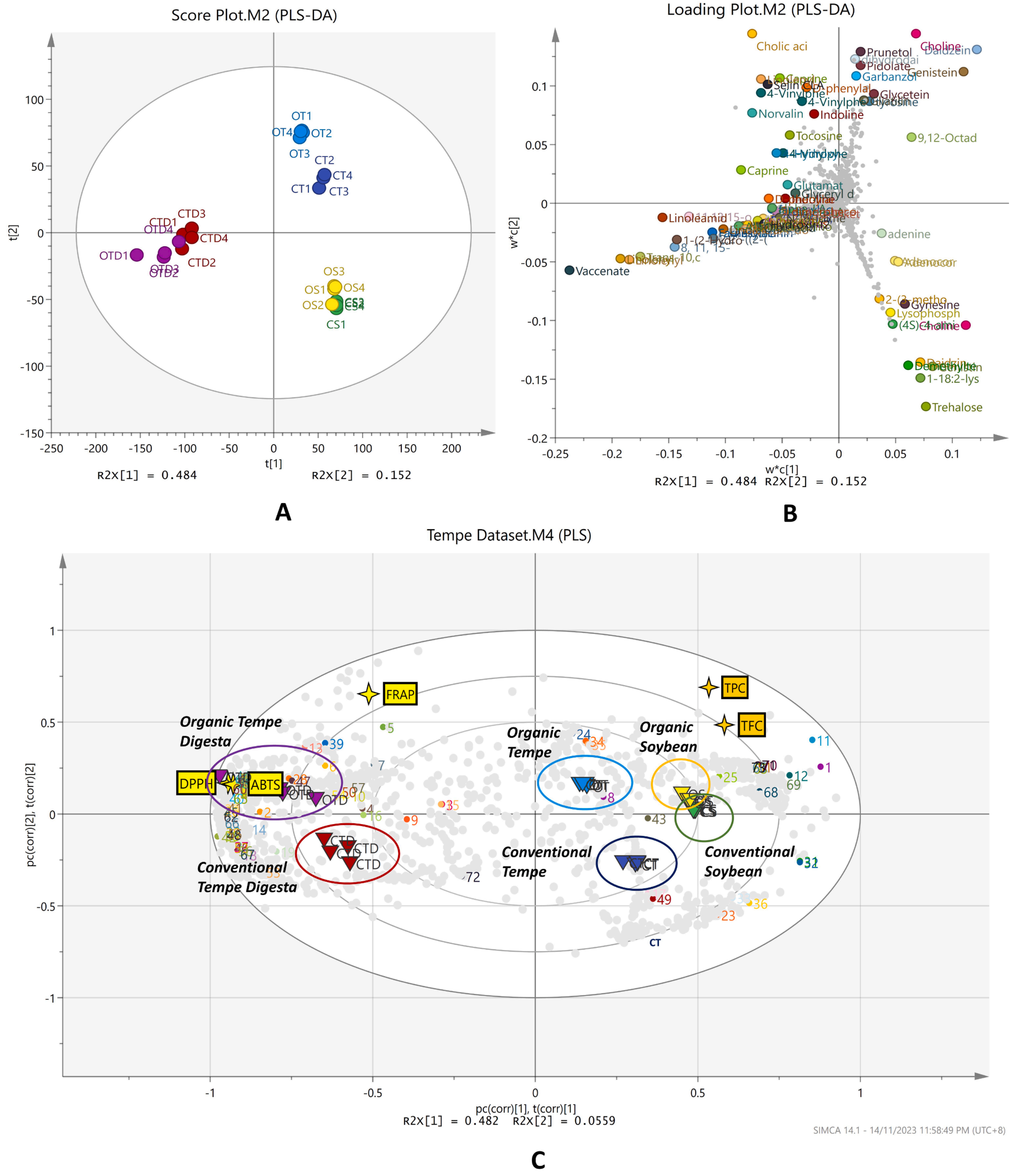

Fig. 3. The MVDA for the normalised data was conducted using SIMCA software version 14.0. **(A)** PLS-DA derived from the LCMS profiles of different sample groups. **(B)** PLS-DA loading plot showing the clustering of metabolites content in different sample groups. **(C)** Biplot from PLS showing the relationship between metabolites as X-variables and biological activities (TPC, TFC and antioxidant activity) as Y-variables.

organic samples of each sample type.

3.6. *Correlation between the metabolites and biological activities of the raw soybean, tempe and tempe digesta samples*

PLS-DA was conducted, as illustrated in Fig. 3, to eliminate numerous extraneous variables and enhance our comprehension of the recognized metabolite patterns in different stages of tempe samples to maximise the differentiation between groups of observations, thereby enhancing classification and prediction abilities. All the samples in the score plot was inside the 95 % Hotelling T2 ellipse with variation of data for PC1 and PC2, 48.4 % and 15.3 %, respectively. The PLS-DA model effectively distinguishes between sample groups ($R^2X = 0.987$, $Q^2Y = 0.901$). From the score plot in Fig. 3A, tempe digesta sample in negative PC1 was shown to be distinctively different from soybean and tempe sample group in positive PC1. Meanwhile, with positive and negative PC2 scores, respectively, the soybean sample group was demonstrated to differ from the tempe sample that was segregated in PC2. No significant difference was observed between conventional and organic samples across soybean, tempe, and digesta groups, similar to the PCA model. The PLS biplot (Fig. 3C) shows a strong correlation between TPC and TFC with soybean samples. Conventional and organic tempe digesta extracts which have the highest DPPH and ABTS free-radical scavenging activities were well separated from the conventional and organic soybean and tempe extracts by PC1 meanwhile FRAP inhibition activity was shown having positively correlated to organic digesta samples which separated by PC2. Although the PLS regression model can observe the correlation between the antioxidant capacity of different tempe samples and their metabolite profiles, the model is not suitable for identifying distinct chemicals that differ between groups.

OPLS-DA in Fig. 4 filters out orthogonal metabolite variables unrelated to categorical variables. It was used to distinguish the metabolite profile and identify metabolite variations among the groups which could enhance classification outcomes. The loading plot displays a data variance of 47 % for PC1 and 14.2 % for PC2. The obtained R^2Y(cum) and Q^2(cum) values of 0.993 and 0.982, respectively, affirm the strong fit and predictability of the OPLS-DA model.

To confirm the model's validity, a permutation test with a sample size of 200 (n = 200) was conducted. The statistical analysis involved filtering the data using specific criteria: VIP>1.5, log2(FC) > 1, and $p < 0.05$ (Table S1). This filtering process helped identify potential characteristic compounds that can be used to distinguish the different antioxidant activities of samples. The results were then visualised in an S-plot, which depicted the covariance (p) against the correlation (pcorr). From the OPLS-DA score plot in Fig. 4A, high antioxidant activity of tempe digesta in positive PC1 was shown to be separated from low antioxidant activity of soybean and tempe in negative PC1 which shows that there is difference in metabolites for these two groups. In Table S1, 34 metabolites have been annotated according to the VIP > 1.5, log2(FC) > 1 and $p < 0.05$. From the 34 metabolites, there are 20 fatty acids and lipids, 8 phenolics and 6 amino acids were annotated and further visualised in S-plot to interpret the importance of variables (features) in discriminating between different classes or groups in MVDA. The S-plot analysis identified key compounds that account for the noteworthy differences between soybean and tempe and tempe digesta (Fig. 4B).

In addition, a correlation study was conducted to observe the statistical correlation between 34 metabolites that were found to be significantly different and the observed patterns of the corresponding phenotypes, including TFC, TPC, DPPH, ABTS, and FRAP (Fig. 5). The Pearson correlation coefficients were computed to analyse the correlation between the relative amounts of the 34 metabolites that substantially discriminate (as shown in Table S2) and their antioxidant activities. Fig. 5 demonstrates that 26 metabolites had positive correlations with antioxidant activity, DPPH and FRAP, whereas 8 metabolites demonstrated negative correlation. Most of the lipids and certain phenolic and amino acid compounds had strong positive correlation with antioxidant activity. Meanwhile, most of the metabolites demonstrated weak positive correlation to TPC and strong negative correlation to TFC.

3.7. *Annotation of isoflavones in the raw soybean, tempe and tempe digesta samples using molecular networking*

In addition to having high protein and carbohydrates, fermented soybeans also include a variety of bioactive substances, such as phenolic acid, sterol, isoflavones, saponins, and flavonoids (do Prado et al., 2022). Isoflavones in soybeans are associated with various health benefits, including maintaining increased antioxidant levels in the body and

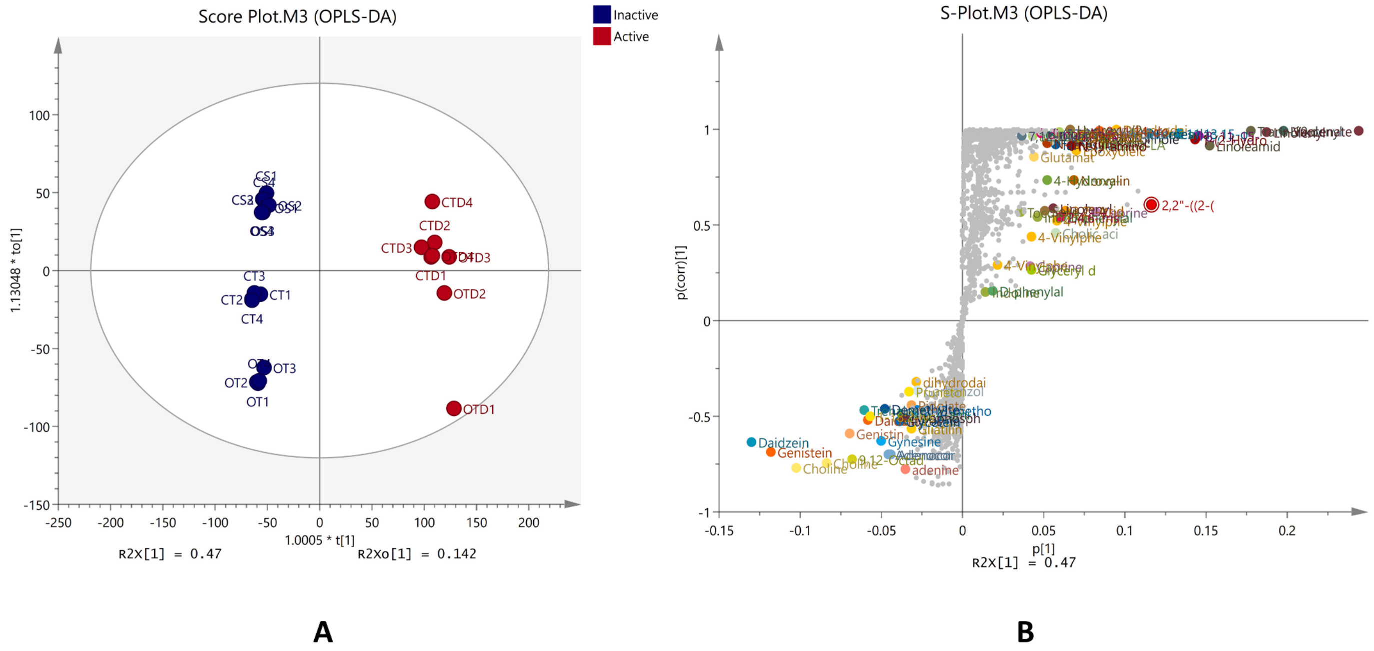

Fig. 4. The MVDA for the normalised data was conducted using SIMCA software version 14.0. **(A)** OPLS-DA derived from the LCMS profiles of different sample groups. **(B)** S-plot showing the metabolites according to the VIP which are responsible for driving the separation of samples.

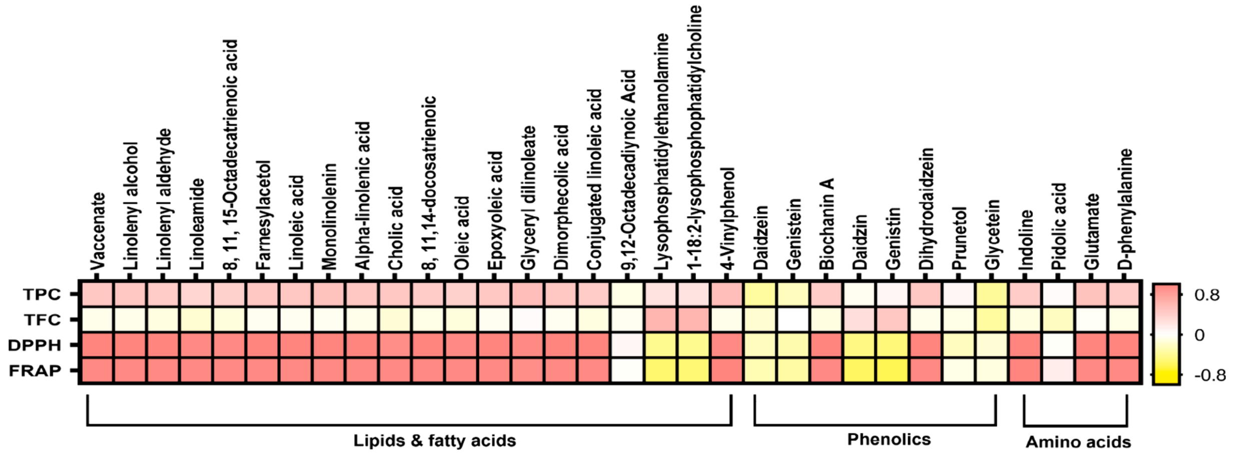

Fig. 5. Graph Pad Prism 9.0 was used to visualise Pearson correlation of significantly different metabolites ($p < 0.05$) with antioxidant assay (DPPH, and FRAP), TPC and TFC of various samples. The values of Pearson's correlation coefficient (r) are indicated by each square with red representing positive ($0 < r < 1$) and yellow representing negative ($-1 < r < 0$) correlations.

supporting cellular health (Brown et al., 2004; DiSilvestro et al., 2006; Messina 2010). Isoflavones can also reduce the risk of cancers such as colon, breast and prostate cancer (Shin et al., 2015; Sivoňová et al., 2019). Food intake that is high in isoflavones such as tempe may help to reduce oxidative stress and inflammation (Valsecchi et al. 2011). Through Feature-based Molecular Networking (FBMN), compounds with similar structure can be clustered and correlated with potential metabolite alterations across various stages (Nothias et al., 2020). This method helps identify different isoflavone forms and possible breakdown products.

Molecular networks with statistical information have been generated by linking the VIP of important features as metadata to compile multivariate data from fingerprinting and feature annotations by molecular networks. A total of 827 features with a VIP value > 1.0 in the OPLS-DA analysis were visualised as orange-coloured nodes in the molecular network (Fig. 6). Nodes with VIP values less than 1 or for which statistical data was not available were maintained as small size and yellow coloured. Clusters of compound families linked to statistically significant features identified during the MVDA were highlighted and spectral similarities were identified through this method. Two clusters were selected which were identified as network clusters of isoflavones. The correlations between these isoflavones and type of samples are visualised in the loading plot in Fig. 3.

3.8. *Heatmap analysis of different isoflavone metabolites*

The heatmap in Fig. S2 presents a comprehensive visualisation of the sample classification, effectively grouping them into distinct classes based on shared attributes. Each row represents an individual sample, while the columns display different features or variables. The intensity of colour in each cell reflects the magnitude of similarity or dissimilarity between samples. Samples belonging to the same class exhibit a strikingly similar pattern, resulting in clusters of cells with high colour intensity. On the other hand, samples from different classes show a discernible contrast in their heatmap patterns, indicating significant differences in their underlying characteristics. Similar to PCA and OPLS-DA, tempe digesta was shown to be in a different group from soybean and tempe with distinctive glycoside isoflavones content. Meanwhile, tempe and tempe digesta samples are shown to be in a different class but less significantly differ from each other. Interestingly, aglycones isoflavones are shown higher in organic tempe digesta while glucosides isoflavones are shown higher in organic and conventional soybeans.

4. Discussions

Food processing and digestion may modify phenolic compounds. In general, the TPC and TFC in tempe samples (both organic and conventional) is shown in decreasing order: raw soybean > tempe > tempe digesta. Taylor (2011) found that heating, rehydration, and leaching phenol chemicals during soaking, boiling, and fermentation significantly reduces phenolic content. Since soaking weakens soybean cell walls, bound polyphenols dissolve in water (Boateng, Verghese, Walker, & Ogutu, 2008). Dehulling lowers TPC by 45 % (Oomah, Cardador-Martínez, & Loarca-Piña, 2005) because the hull contains more phenolic chemicals than the cotyledon (Pastor-Cavada et al., 2009). Similarly, fermentation leads to TPC reduction in 48-hour fermented conventional and organic tempe (Athaillah et al., 2019; Mastura et al., 2017; Raob, 2014). Organic samples showed higher TPC and TFC than conventional samples before and after gastrointestinal digestion. This is due to the absence of synthetic fertilisers and pesticides in organic farming, which can enhance polyphenol synthesis (Winter & Davis, 2006; Mastura et al., 2017). In addition, the TFC is higher in soybeans compared to tempe and digested tempe. This may be because the isoflavones in soybeans are in the form of larger molecules such as glycosides, acetylglycosides, and malonylglycosides, with a lower proportion existing as aglycones. During tempe fermentation, isoflavone glucoside conjugates undergo conversion into aglycones by the action of β-glucosidase (Hati, Vij, Singh, & Mandal, 2015). TPC and TFC decrease after *in vitro* gastrointestinal digestion due to possible binding of the polyphenol-pepsin complex via hydrogen bonding, van der Waals interactions, and pancreatin-induced hydrolysis rate reduction (Zhou, Hu, Tan, Zhang, & Mcclements, 2021). Our heatmap analysis also revealed that aglycone isoflavones were higher in organic tempe digesta, while glucoside isoflavones were higher in both organic and conventional soybeans. Nevertheless, there are also conflicting findings in the literature. Several studies showed that the TPC of the fermented soybeans extracts were significantly higher ($p < 0.05$) than the unfermented soybean extracts (Toor, Kaur, Sahota, & Kaur, 2021; Xiao et al., 2016). Polyphenol bioaccessibility is affected by their physicochemical qualities, food matrix, interaction with other components, and cofactors or inhibitors (Wojtunik-Kulesza et al., 2020). The content of phenolic chemicals in the food matrix may explain these discrepancies in outcomes.

Many studies have discussed the increase of antioxidants activity in tempe in comparison to soybean. A study reported the increase in scavenging activity up to 24.56 % in tempe in comparison to soybean (Barus et al., 2019). Another study reported a 10.71 % antioxidant activity increase in tempe in comparison to soybean (Tjandra et al., 2021).

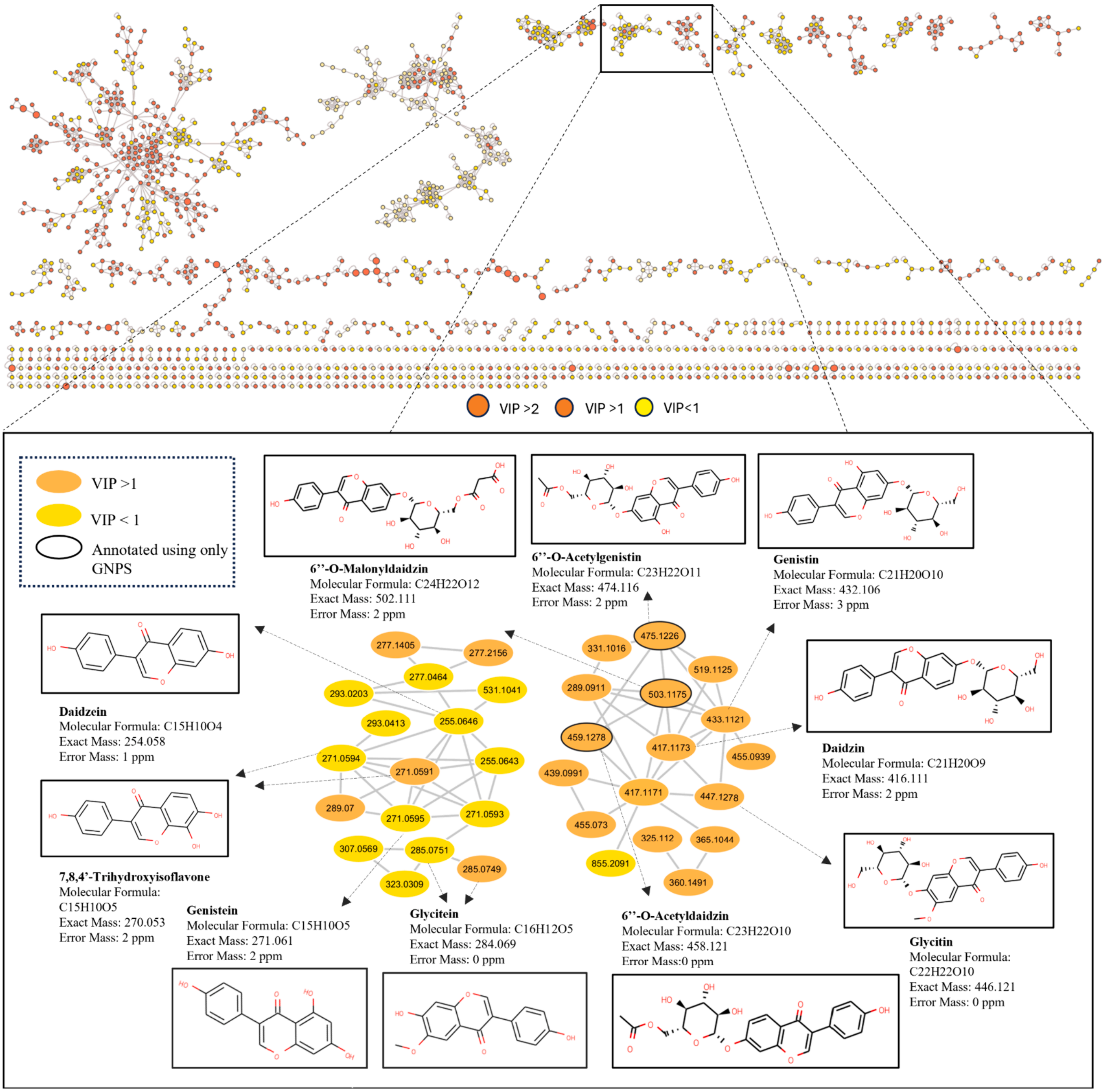

Fig. 6. Clusters of isoflavones were chosen from the statistically generated molecular network. Nodes with VIP values exceeding 1 (depicted in orange) from the OPLS model were incorporated into the network, with node size and colour indicating their significance. Orange nodes denote VIP values above 1, while yellow nodes signify values below 1. Nodes with VIP values above 2 are depicted as larger nodes. Metabolites with black borders are exclusively identified through the GNPS database, whereas those without borders are annotated using both GNPS and SIRIUS databases.

Similarly, in this study, FRAP assay shows a significant increase in FRAP inhibition in tempe compared to soybean for both conventional and organic samples. Previous study has discussed the *in vitro* digestibility of fermented soybean known as natto specifically focusing on the breakdown of protein where fermented soybeans showed significantly higher antioxidant activity in comparison to soaked and boiled soybean after intestinal digestion (Ketnawa & Ogawa, 2021). Another study demonstrates the increase of antioxidant level of fermented black bean tempe is up to 0.63 times after *in vitro* digestion (Wang et al., 2022). These study results aligned with the increase in antioxidant activity in tempe digesta compared to tempe for both conventional and organic samples.

When comparing conventional and organic samples, there is a significant difference where organic samples exhibit higher antioxidant activity compared to conventional samples particularly organic tempe digesta having the highest antioxidant activity. There is debate in the research over whether organic and non-organic foods differ significantly in terms of nutrition and biological effects. Some studies report a significantly higher antioxidant activity in organic crops compared to conventional crops while others report no difference in antioxidant between conventional crops and organic crops (Balisteiro, Rombaldi, & Genovese, 2013; Barański et al., 2014). Organic crops are believed to contain higher antioxidants as they consist of higher natural bioactive compounds produced as the result of exposure to stressful conditions (Winter & Davis, 2006). However, there is still a lack of study on organic

food in regard to its antioxidant capacity in comparison to conventional-based food.

Cell viability decreases in all extract samples, however the IC_{50} value showed no cytotoxicity effect. Previously, tempe has been studied with the concentration of sample treatment of 4 mg per 200 µl of acetone solvents for its cytotoxicity targeting human breast adenocarcinoma cell lines (MCF-7) with various fermentation periods and the result shows moderate to high cytotoxicity for 48-hour fermentation (Athaillah et al., 2019; Devi et al., 2021). Another study found significant cytotoxicity in HCT116 cells with enzyme-hydrolyzable bound (EhBP) phenolic tempe extracts at 100 µg/mL (Ahnan-Winarno et al., 2021). The lack of cytotoxic effects observed in the study may be related to the concentration employed which might be insufficient to induce cytotoxic effects and the choice of solvent which may impact the extraction efficiency of bioactive compounds, consequently affecting the cytotoxic potential. Other factors include cell quenching, heterogeneity in culture medium compositions, variances in passage number, and contamination could contribute to inherent biases in cell metabolomics (Yusof et al., 2021).

Findings from PCA show that soybeans are higher in more complex isoflavone glycosides, including notable compounds such as genistin and daidzin, as well as primary metabolites like trehalose. These glycosides undergo conversion into aglycones during fermentation, most likely as a result of bacteria that produce the enzyme β-glucosidase (Li et al., 2021). Using the sugar groups from glycosides, this enzyme hydrolyses glycosidic bonds to create organic acids such as lactic and acetic acids (Zhao et al., 2023). Aglycones such as genistein and daidzein are produced by this method. Furthermore, pancreatic lipases' breakdown of soybean triglycerides during *in vitro* gastrointestinal digestion produces fatty acids, which are abundant in tempe digesta (Martin and Freedman, 2018; Zhu et al., 2021).

Results from the PLS biplot indicate a strong correlation between TPC and TFC with conventional and organic soybean samples. From this correlation, in comparison to tempe and tempe digesta samples, it can be concluded that TPC and TFC of soybean samples is higher, possibly due to the phenolic and flavonoid chemicals that have not undergone further degradation into smaller compounds. During the fermentation process used to make tempe, some of these phenolic and flavonoid compounds may be broken down or transformed, leading to a decrease in TPC compared to the original soybeans. From the 34 metabolites, there are 20 fatty acids and lipids, 8 phenolics and 6 amino acids were annotated.

Based on the Pearson correlation analysis, lipid, phenolic, and amino acid metabolites accounted for 26 metabolites showing positive correlations with antioxidant activity, DPPH, and FRAP assays. Lipids, such as polyunsaturated fatty acids (PUFAs) like linolenyl aldehyde and conjugated linoleic acid (CLA), are involved in antioxidant activity due to their ability to act as electron donors, neutralizing free radicals and preventing cellular damage. Compounds such as 8,11,15-Octadecatrienoic acid and 11,13,15-octadecatrienoic acid, both forms of omega-3 fatty acids, contribute to antioxidant defense by mitigating oxidative stress (Yuan et al., 2014). Additionally, linoleic acid and oleic acid, common dietary fats, have roles in antioxidant activity, with other lipid metabolites like prunetol and 12,15-Octadecadiynoic acid also studied for their potential effects.

Phenolic isoflavones in soybeans are associated with increased antioxidant levels and supporting cellular health (Brown et al., 2004; DiSilvestro et al., 2006; Messina, 2010). Biochanin A, strongly correlated with DPPH and FRAP antioxidant activity, is recognized for its potent antioxidant properties, effectively scavenging reactive oxygen species (ROS) and increasing levels of catalase and superoxide dismutase (Raheja et al., 2018). Dihydrodaidzein, derived from daidzein through fermentation involving lactic acid bacteria, enhances the production of equol and 5-OH-equol in equol producers (Langa et al., 2023). Amino acids such as tyrosine and glutamate also demonstrate significant antioxidant roles. Tyrosine and glutamate are precursors for important biological compounds, illustrating the antioxidant capabilities of amino acids (Slominski et al., 2012; Sedlak et al., 2019).

Three GNPS database-identified acetyl isoflavones and glycoside isoflavones such daidzin, glycitin, and genistin, along with their acetyl derivatives, may affect bioavailability and biological activity. The molecular network linkages between these chemicals reveal structural similarities and enzyme changes. FBMN better identified isomers with similar molecular weights and formulae than SIRIUS annotation. The identification of 7,8,4′-trihydroxyisoflavone highlights its role as an intermediary or metabolite in isoflavone pathways, with health-promoting qualities such as antioxidant and anti-atopic actions (Ko et al., 2019).

The heatmap analysis revealed that aglycone isoflavones were higher in organic tempe digesta, while glucoside isoflavones were higher in both organic and conventional soybeans. Aglycone isoflavones, easily absorbed in the small intestine, are more bioavailable due to their non-sugar form (Kim, 2021). Glycoside isoflavones, linked to sugar molecules, undergo enzymatic cleavage in the gastrointestinal tract to transform into aglycone forms before absorption. During fermentation, glycoside isoflavones hydrolyze, becoming aglycone isoflavones. Further breakdown of isoflavones, such as daidzein into dihydrodaidzein, equol, and O-desmethylangolensin by gut bacteria, occurs in the gastrointestinal tract (Takagi et al., 2015).

5. Conclusion

In conclusion, the results suggest that metabolites undergo modifications throughout the food processing and digestion of tempe in our digestive systems. The findings demonstrated that the overall content of flavonoids and phenolics significantly decreased in tempe from soybean. Organic tempe had higher levels of phenolic and flavonoid content than conventional tempe. This is likely because synthetic fertilizers and pesticides are not used in organic farms, which can boost polyphenol synthesis. However, further study is needed to investigate the effect of synthetic fertilizers and pesticides on the phenolic and flavonoid content. Antioxidants assays also have demonstrated that tempe has greater antioxidant capacity in comparison to soybean, particularly after *in-vitro* digestion. Analyses of the chemical components in tempe revealed clear differences at various stages, with specific compounds like lipids, amino acids, and isoflavones contributing to these distinctions. Moreover, statistical analyses demonstrated a strong correlation between the specific compounds present in digested organic tempe after digestion and the antioxidant activity. Overall, the findings highlight the unique chemical profiles and variety of bioactive qualities that tempe exhibits post-digestion, providing insight into its nutritional benefits.

Declaration of generative AI and AI-assisted technologies in the writing process

The authors used ChatGPT 3.5 in order to assist in generating and refining content, facilitating the exploration of language nuances, and enhancing overall communication clarity while this work was being prepared. Following the use of this tool/service, the authors took full responsibility for the content in publication, reviewed and edited it as required.

CRediT authorship contribution statement

Nurul Syahidah Mio Asni: Writing – review & editing, Writing – original draft, Visualization, Validation, Software, Methodology, Investigation, Formal analysis, Data curation. **Reggie Surya:** Writing – review & editing, Supervision. **Norazlan Mohmad Misnan:** Writing – review & editing, Methodology, Formal analysis. **Seng Joe Lim:** Writing – review & editing, Supervision, Resources, Methodology, Conceptualization. **Norzila Ismail:** Writing – review & editing, Supervision, Resources, Methodology, Conceptualization. **Shahrul Razid Sarbini:** Writing – review & editing, Supervision, Resources, Methodology, Conceptualization. **Nurkhalida Kamal:** Writing – review & editing,

Supervision, Resources, Methodology, Funding acquisition, Conceptualization.

Declaration of competing interest

The authors declare that they have no known competing financial interests or personal relationships that could have appeared to influence the work reported in this paper.

Data availability

Data will be made available on request.

Acknowledgement

The authors extend their appreciation to the Universiti Kebangsaan Malaysia, Malaysia for funding this work through a Fundamental Research Grant Scheme under grant number FRGS/1/2022/WAB13/UKM/02/1. We would like to thank Dr. Pouya Hassandarvish, a senior lecturer at Universiti Malaya (UM), for providing the HCT-116 cell lines for this study. The authors also would like to thank Tan Hui Yan from Universiti Putra Malaysia (UPM), Bintulu and also Nor Amalia Binti Nazri and Taif Al Qaisi Binti Kareem from Universiti Sains Malaysia (USM), Kubang Kerian for their assistance and the valuable training provided.

Appendix A. Supplementary material

Supplementary data to this article can be found online at https://doi.org/10.1016/j.foodres.2024.114951.

References

Ahnan-Winarno, A. D., Cordeiro, L., Winarno, F. G., Gibbons, J., & Xiao, H. (2021). Tempeh: A semicentennial review on its health benefits, fermentation, safety, processing, sustainability, and affordability. *Comprehensive Reviews in Food Science and Food Safety, 20*(2), 1717–1767. https://doi.org/10.1111/1541-4337.12710

Astuti, M., Meliala, A., Dalais, F., & Wahlqvist, M. (2000). Tempe, a nutritious and healthy food from Indonesia. *Asia Pacific Journal of Clinical Nutrition, 9*, 322–325. https://doi.org/10.1046/j.1440-6047.2000.00176.x

Athaillah, Z. A., Muzdalifah, D., Lestari, A., Devi, A. F., Udin, L. Z., Artanti, N., & Lioe, H. N. (2019). Phenolic compound profile and functionality of aqueous overripe tempe extracts. *Current Research in Nutrition and Food Science, 7*(2), 382–392. https://doi.org/10.12944/CRNFSJ.7.2.08

Athilah, F. A. F., Afnani, A., Nurul, A. H. Z., & Noor, A. M. (2020). Total phenolic, total flavonoids content and antioxidant activity of Mangifera sp. leaf extracts. *Journal of Agrobiotechnology, 11*(1S). https://doi.org/10.37231/jab.2020.11.1s.236

Balisteiro, D. M., Rombaldi, C. V., & Genovese, M. I. (2013). Protein, isoflavones, trypsin inhibitory and *in vitro* antioxidant capacities: Comparison among conventionally and organically grown soybeans. *Food Research International, 51*(1), 8–14. https://doi.org/10.1016/j.foodres.2012.11.015

Barański, M., Średnicka-Tober, D., Volakakis, N., Seal, C., Sanderson, R., Stewart, G. B., … Leifert, C. (2014). Higher antioxidant and lower cadmium concentrations and lower incidence of pesticide residues in organically grown crops: A systematic literature review and meta-analyses. *British Journal of Nutrition, 112*(50), 794–811. https://doi.org/10.1017/S0007114514001366

Barus, T., Titarsole, N. N., Mulyono, N., & Prasasty, V. D. (2019). Tempeh antioxidant activity using DPPH method: Effects of fermentation, processing, and microorganisms. *Journal of Food Engineering and Technology, 8*(2), 75–80. https://doi.org/10.32732/jfet.2019.8.2.75

Boateng, J., Verghese, M., Walker, L. T., & Ogutu, S. (2008). Effect of processing on antioxidant contents in selected dry beans (*Phaseolus* spp. L.). *LWT, 41*(9), 1541–1547. https://doi.org/10.1016/j.lwt.2007.11.025

Bristy, A. T., Islam, T., Ahmed, R., Hossain, J., Reza, H. M., & Jain, P. (2022). Evaluation of total phenolic content, HPLC analysis, and antioxidant potential of three local varieties of mushroom: A comparative study. *International Journal of Food Science, 2022*. https://doi.org/10.1155/2022/3834936

Brown, E. C., DiSilvestro, R. A., Babaknia, A., & Devor, S. T. (2004). Soy versus whey protein bars: Effects on exercise training impact on lean body mass and antioxidant status. *Nutrition Journal, 3*. https://doi.org/10.1186/1475-2891-3-22

Chong, S. G., Ismail, I. S., Ahmad Azam, A., Tan, S. J., Shaari, K., & Tan, J. K. (2023). Nuclear magnetic resonance spectroscopy and liquid chromatography–mass spectrometry metabolomics studies on non-organic soybeans versus organic soybeans (Glycine max), and their fermentation by *Rhizopus oligosporus*. *Journal of the Science of Food and Agriculture, 103*(6), 3146–3156. https://doi.org/10.1002/jsfa.12355

Devi, A. F., Muzdalifah, D., Athaillah, Z. A., Lioe, H. N., & Artanti, N. (2021). Isoflavones and bioactivities in over-fermented tempeh extracts. *Jurnal Kimia Sains Dan Aplikasi, 24*(7), 244–251. https://doi.org/10.14710/jksa.24.7.244-251

DiSilvestro, R. A., Mattern, C., Wood, N., & Devor, S. T. (2006). Soy protein intake by active young adult men raises plasma antioxidant capacity without altering plasma testosterone. *Nutrition Research, 26*(2), 92–95. https://doi.org/10.1016/j.nutres.2005.12.002

do Prado, F. G., Pagnoncelli, M. G. B., de Melo Pereira, G. V., Karp, S. G., & Soccol, C. R. (2022). Fermented soy products and their potential health benefits: A review. *Microorganisms, 10*(8). https://doi.org/10.3390/microorganisms10081606. MDPI.

Dührkop, K., Fleischauer, M., Ludwig, M., Aksenov, A. A., Melnik, A. V., Meusel, M., Dorrestein, P. C., Rousu, J., & Böcker, S. (2019). SIRIUS 4: A rapid tool for turning tandem mass spectra into metabolite structure information. *Nature Methods, 16*(4), 299–302. https://doi.org/10.1038/s41592-019-0344-8

Faliq, W. S., Binti, A., & Ibrahim, W. (2020). *Phytochemical characterization, induction of apoptosis and activation of natural killer (NK) cells by Abrus precatorius leaves extract on human breast cancer cell line*.

Hati, S., Vij, S., Singh, B. P., & Mandal, S. (2015). β-Glucosidase activity and bioconversion of isoflavones during fermentation of soymilk. *Journal of the Science of Food and Agriculture, 95*(1), 216–220. https://doi.org/10.1002/jsfa.6743

Kanissery, R., Gairhe, B., Kadyampakeni, D., Batuman, O., & Alferez, F. (2019). Glyphosate: Its environmental persistence and impact on crop health and nutrition. *Plants, 8*(11), 499. https://doi.org/10.3390/plants8110499

Kasim, N., Afzan, A., Manshoor, N., & Ismail, N. H. (2023). Quantification of vitexin and isovitexin in seven varieties of Ficus deltoidea in Peninsular Malaysia. *Malaysian Journal of Chemistry, 25*(1).

Ketnawa, S., & Ogawa, Y. (2021). *In vitro* protein digestibility and biochemical characteristics of soaked, boiled and fermented soybeans. *Scientific Reports, 11*(1). https://doi.org/10.1038/s41598-021-93451-x

Kim, I. S. (2021). Current perspectives on the beneficial effects of soybean isoflavones and their metabolites for humans. *Antioxidants, 10*(7). https://doi.org/10.3390/ANTIOX10071064/S1

Klus, K., Börger-Papendorf, G., & Barz, W. (1993). Formation of 6, 7, 4′-trihydroxyisoflavone (factor 2) from soybean seed isoflavones by bacteria isolated from tempe. *Phytochemistry, 34*(4), 979–981.

Ko, Y. H., Kim, S. K., Kwon, S. H., Seo, J. Y., Lee, B. R., Kim, Y. J., Hur, K. H., Kim, S. Y., Lee, S. Y., & Jang, C. G. (2019). 7,8,4'-trihydroxyisoflavone, a metabolized product of daidzein, attenuates 6-hydroxydopamine-induced neurotoxicity in SH-SY5Y cells. *Biomolecules and Therapeutics, 27*(4), 363–372. https://doi.org/10.4062/biomolther.2018.211

Langa, S., Peirotén, Á., Curiel, J. A., de la Bastida, A. R., & Landete, J. M. (2023). Isoflavone metabolism by lactic acid bacteria and its application in the development of fermented soy food with beneficial effects on human health. *Foods, 12*(6), 1293. https://doi.org/10.3390/foods12061293

Li, C., Xu, T., Liu, X. W., Wang, X., & Xia, T. (2021). The expression of β-glucosidase during natto fermentation increased the active isoflavone content. *Food Bioscience, 43*. https://doi.org/10.1016/j.fbio.2021.101286

Limanjaya, E. C., Subali, D., & Yanti, Y. (2022). The anti-Alzheimer compounds from tempeh oil in LPS-induced neuronal Schwann cells. *Journal of Ethnic Foods, 9*(1). https://doi.org/10.1186/s42779-022-00163-2

Macintyre, L., Zhang, T., Viegelmann, C., Martinez, I. J., Cheng, C., Dowdells, C., Abdelmohsen, U. R., Gernert, C., Hentschel, U., & Edrada-Ebel, R. A. (2014). Metabolomic tools for secondary metabolite discovery from marine microbial symbionts. *Marine Drugs, 12*(6), 3416–3448. https://doi.org/10.3390/md12063416

Martin, C. R., & Freedman, S. D. (2018). Lipid and fatty acid delivery in the preterm infant: Challenges and lessons learned from other critically ill populations. In *Gastroenterology and nutrition: Neonatology questions and controversies* (pp. 29–41). Elsevier. https://doi.org/10.1016/B978-0-323-54502-0.00003-7.

Mastura, H., Hasnah, Y., & Dang, H. (2017). Total phenolic content and antioxidant capacity of beans: Organic vs inorganic abstract. *International Food Research Journal, 4*(2).

Meftaul, I. M., Venkateswarlu, K., Dharmarajan, R., Annamalai, P., Asaduzzaman, M., Parven, A., & Megharaj, M. (2020). Controversies over human health and ecological impacts of glyphosate: Is it to be banned in modern agriculture? *Environmental Pollution, 263*, Article 114372. https://doi.org/10.1016/j.envpol.2020.114372

Messina, M. (2010). A brief historical overview of the past two decades of soy and isoflavone research. *Journal of Nutrition, 140*(7). https://doi.org/10.3945/jn.109.118315

Mohamed Idris, Z., Jamal, P., Zahangir Alam, M., Haseeb, A., Mai Sci, C. J., Haseeb Ansari, A., & Ahmad Barkat, A. (2015). Phenolics production from a novel substrate palm oil mill effluent by Aspergillus niger IBS-103ZA: Evaluation of fermentation conditions and antioxidant activity production of cellulolytic enzymes from industrial and agricultural wastes. *Chiang Mai Journal of Science, 42*(1). http://epg.science.cmu.ac.th/ejournal/.

Morales, G., & Paredes, A. (2014). Antioxidant activities of Lampaya medicinalis extracts and their main chemical constituents. *BMC Complementary and Alternative Medicine, 14*. https://doi.org/10.1186/1472-6882-14-259

Mulet-Cabero, A. I., Egger, L., Portmann, R., Ménard, O., Marze, S., Minekus, M., Le Feunteun, S., Sarkar, A., Grundy, M. M. L., Carrière, F., Golding, M., Dupont, D., Recio, I., Brodkorb, A., & Mackie, A. (2020). A standardised semi-dynamic: *In vitro* digestion method suitable for food-an international consensus. *Food and Function, 11*(2), 1702–1720. https://doi.org/10.1039/c9fo01293a

Nothias, L. F., Petras, D., Schmid, R., Dührkop, K., Rainer, J., Sarvepalli, A., … Dorrestein, P. C. (2020). Feature-based molecular networking in the GNPS analysis environment. *Nature Methods, 17*(9), 905–908. https://doi.org/10.1038/s41592-020-0933-6

Nowak, D., & Jakubczyk, E. (2020). The freeze-drying of foods ⇔ the characteristic of the process course and the effect of its parameters on the physical properties of food materials. *Foods, 9*(10), 1488. https://doi.org/10.3390/foods9101488
Oomah, B. D., Cardador-Martínez, A., & Loarca-Piña, G. (2005). Phenolics and antioxidative activities in common beans (*Phaseolus vulgaris* L). *Journal of the Science of Food and Agriculture, 85*(6), 935–942. https://doi.org/10.1002/jsfa.2019
Pastor-Cavada, E., Juan, R., Pastor, J. E., Alaiz, M., & Vioque, J. (2009). Antioxidant activity of seed polyphenols in fifteen wild Lathyrus species from South Spain. *LWT, 42*(3), 705–709. https://doi.org/10.1016/j.lwt.2008.10.006
Raheja, S., Girdhar, A., Lather, V., & Pandita, D. (2018). Biochanin A: A phytoestrogen with therapeutic potential. In *Trends in Food Science and Technology* (Vol. 79, pp. 55–66). Elsevier Ltd.. https://doi.org/10.1016/j.tifs.2018.07.001
Raob, N. (2014). Changes in macronutrient, total phenolic and anti-nutrient contents during preparation of tempeh. *Journal of Nutrition & Food Sciences, 4*(03). https://doi.org/10.4172/2155-9600.1000265
Rocchetti, G., Senizza, B., Giuberti, G., Montesano, D., Trevisan, M., & Lucini, L. (2020). Metabolomic study to evaluate the transformations of extra-virgin olive oil's antioxidant phytochemicals during *in vitro* gastrointestinal digestion. *Antioxidants, 9* (4). https://doi.org/10.3390/antiox9040302
Rutz, A., Dounoue-Kubo, M., Ollivier, S., Bisson, J., Bagheri, M., Saesong, T., Ebrahimi, S. N., Ingkaninan, K., Wolfender, J. L., & Allard, P. M. (2019). Taxonomically informed scoring enhances confidence in natural products annotation. *Frontiers in Plant Science, 10*. https://doi.org/10.3389/fpls.2019.01329
Santana Andrade, J. K., Chagas Barros, R. G., Pereira, U. C., Nogueira, J. P., Gualberto, N. C., Santos de Oliveira, C., Shanmugam, S., & Narain, N. (2022). Bioaccessibility of bioactive compounds after *in vitro* gastrointestinal digestion and probiotics fermentation of Brazilian fruits residues with antioxidant and antidiabetic potential. *LWT, 153*. https://doi.org/10.1016/j.lwt.2021.112469
Sedlak, T. W., Paul, B. D., Parker, G. M., Hester, L. D., Snowman, A. M., Taniguchi, Y., Kamiya, A., Snyder, S. H., & Sawa, A. (2019). The glutathione cycle shapes synaptic glutamate activity. *Proceedings of the National Academy of Sciences of the United States of America, 116*(7), 2701–2706. https://doi.org/10.1073/pnas.1817885116
Schmid, R., Heuckeroth, S., Korf, A., Smirnov, A., Myers, O., Dyrlund, T. S., Bushuiev, R., Murray, K. J., Hoffmann, N., Lu, M., Sarvepalli, A., Zhang, Z., Fleischauer, M., Dührkop, K., Wesner, M., Hoogstra, S. J., Rudt, E., Mokshyna, O., Brungs, C., … Pluskal, T. (2023). Integrative analysis of multimodal mass spectrometry data in MZmine 3. *Nature Biotechnology, 41*(4), 447–449. https://doi.org/10.1038/s41587-023-01690-2
Sembiring, E. N., Elya, B., & Sauriasari, R. (2018). Phytochemical screening, total flavonoid and total phenolic content and antioxidant activity of different parts of Caesalpinia bonduc (L.) Roxb. *Pharmacognosy Journal, 10*(1), 123–127. https://doi.org/10.5530/pj.2018.1.22
Shin, A., Lee, J., Lee, J., Park, M. S., Park, J. W., Park, S. C., Oh, J. H., & Kim, J. (2015). Isoflavone and soyfood intake and colorectal cancer risk: A case-control study in Korea. *PLoS One, 10*(11). https://doi.org/10.1371/journal.pone.0143228
Sivoňová, M. K., Kaplán, P., Tatarková, Z., Lichardusová, L., Dušenka, R., & Jurečeková, J. (2019). Androgen receptor and soy isoflavones in prostate cancer (Review). *In Molecular and Clinical Oncology, 2*, 191–204. https://doi.org/10.3892/mco.2018.1792
Slominski, A., Zmijewski, M. A., & Pawelek, J. (2012). L-tyrosine and L-dihydroxyphenylalanine as hormone-like regulators of melanocyte functions. *Pigment Cell and Melanoma Research, 25*(1), 14–27. https://doi.org/10.1111/j.1755-148X.2011.00898.x
Soumya, N. P. P., Mini, S., Sivan, S. K., & Mondal, S. (2021). Bioactive compounds in functional food and their role as therapeutics. *Bioactive Compounds in Health and Disease, 4*(3), 24–39. https://doi.org/10.31989/bchd.v4i3.786
Takagi, A., Kano, M., & Kaga, C. (2015). Possibility of breast cancer prevention: Use of soy isoflavones and fermented soy beverages produced using probiotics. *International Journal of Molecular Sciences, 16*(5), 10907–10920. https://doi.org/10.3390/ijms160510907
Taylor, J. (2011). *The functionality of tempeh addition to beef patties*.
Tjandra, L., Setiawan, B., Ishartadiati, K., Utami, S. L., & Widjaja, J. H. (2021). The effects of tempe extract on the oxidative stress marker and lung pathology in tuberculosis Wistar rat. *Russian Open Medical Journal, 10*(4). https://doi.org/10.15275/rusomj.2021.0412
Toor, B. S., Kaur, A., Sahota, P. P., & Kaur, J. (2021). Antioxidant Potential, Antinutrients, Mineral Composition and FTIR Spectra of Legumes Fermented with Rhizopus oligosporus. *Food Technology and Biotechnology, 59*(4), 530–542. https://doi.org/10.17113/ftb.59.04.21.7319
Valsecchi, A. E., Franchi, S., Panerai, A. E., Rossi, A., Sacerdote, P., & Colleoni, M. (2011). The soy isoflavone genistein reverses oxidative and inflammatory state, neuropathic pain, neurotrophic and vasculature deficits in diabetes mouse model. *European Journal of Pharmacology, 650*(2–3), 694–702. https://doi.org/10.1016/j.ejphar.2010.10.060
Vazquez-Aguilar, A., Sanchez-Rodriguez, E., Rodriguez-Perez, C., Rangel-Huerta, O. D., & Mesa, M. D. (2023). Metabolomic-based studies of the intake of virgin olive oil: A comprehensive review. *Metabolites, 13*(4), 472. https://doi.org/10.3390/metabo13040472
Wang, K., Gao, Y., Zhao, J., Wu, Y., Sun, J., Niu, G., Zuo, F., & Zheng, X. (2022). Effects of *in vitro* digestion on protein degradation, phenolic compound release, and bioactivity of black bean tempeh. *Frontiers in Nutrition, 9*. https://doi.org/10.3389/fnut.2022.1017765
Wang, M., Carver, J. J., Phelan, V. V., Sanchez, L. M., Garg, N., Peng, Y., Nguyen, D. D., Watrous, J., Kapono, C. A., Luzzatto-Knaan, T., Porto, C., Bouslimani, A., Melnik, A. V., Meehan, M. J., Liu, W. T., Crüsemann, M., Boudreau, P. D., Esquenazi, E., Sandoval-Calderón, M., … Bandeira, N. (2016). Sharing and community curation of mass spectrometry data with Global Natural Products Social Molecular Networking. *Nature Biotechnology, 34*(8), 828–837. https://doi.org/10.1038/nbt.3597
Winter, C. K., & Davis, S. F. (2006). Organic foods. *Journal of Food Science, 71*(9). https://doi.org/10.1111/j.1750-3841.2006.00196.x
Wojtunik-Kulesza, K., Oniszczuk, A., Oniszczuk, T., Combrzyński, M., Nowakowska, D., & Matwijczuk, A. (2020). Influence of in vitro digestion on composition, bioaccessibility and antioxidant activity of food polyphenols—A non-systematic review. *Nutrients, 12*(5), 1401. https://doi.org/10.3390/nu12051401
Wu, S. K., & Hasnah, H. (2018). Nutrient contents in tempe produced from five cottage industries in Selangor, Malaysia. *Jurnal Sains Kesihatan Malaysia, 16*(1), 1–6. https://doi.org/10.17576/JSKM-2018-1601-01
Xiao, Y., Fan, J., Chen, Y., Rui, X., Zhang, Q., & Dong, M. (2016). Enhanced total phenolic and isoflavone aglycone content, antioxidant activity and DNA damage protection of soybeans processed by solid state fermentation with Rhizopus oligosporus RT-3. *RSC Advances, 6*(35), 29741–29756. https://doi.org/10.1039/C6RA00074F
Yu, X., Guo, L., Jiang, G., Song, Y., & Muminov, M. A. (2018). Advances of organic products over conventional productions with respect to nutritional quality and food security. *Acta Ecologica Sinica, 38*(1), 53–60. https://doi.org/10.1016/j.chnaes.2018.01.009
Yuan, G. F., Chen, X. E., & Li, D. (2014). Conjugated linolenic acids and their bioactivities: A review. *Food & Function, 5*(7), 1360–1368.
Yusof, H. M., Ab-Rahim, S., Wan Ngah, W. Z., Nathan, S., Jamal, A. A. R., & Mazlan, M. (2021). Metabolomic characterization of colorectal cancer cell lines highlighting stage-specific alterations during cancer progression. *BioImpacts: BI, 11*(2), 147–156. https://doi.org/10.34172/BI.2021.22
Zhao, S., Sai, Y., Liu, W., Zhao, H., Bai, X., Song, W., Zheng, Y., & Yue, X. (2023). Flavor characterization of traditional fermented soybean pastes from Northeast China and Korea. *Foods, 12*(17). https://doi.org/10.3390/foods12173294
Zhou, H., Hu, Y., Tan, Y., Zhang, Z., & McClements, D. J. (2021). Digestibility and gastrointestinal fate of meat versus plant-based meat analogs: An in vitro comparison. *Food Chemistry, 364*, Article 130439.
Zhu, G., Fang, Q., Zhu, F., Huang, D., & Yang, C. (2021). Structure and function of pancreatic lipase-related protein 2 and its relationship with pathological states. *Frontiers in Genetics, 12*, Article 693538. https://doi.org/10.3389/fgene.2021.693538
